# Molecular phylogenetics and character evolution of morphologically diverse groups, *Dendrobium* section *Dendrobium* and allies

**DOI:** 10.1093/aobpla/plu045

**Published:** 2014-08-07

**Authors:** Tomoko Takamiya, Pheravut Wongsawad, Apirada Sathapattayanon, Natsuko Tajima, Shunichiro Suzuki, Saki Kitamura, Nao Shioda, Takashi Handa, Susumu Kitanaka, Hiroshi Iijima, Tomohisa Yukawa

**Affiliations:** 1 School of Pharmacy, Nihon University, Funabashi, Chiba 274-8555, Japan; 2 Institute of Agriculture and Forestry, University of Tsukuba, Tsukuba, Ibaraki 305-8572, Japan; 3 Faculty of Science, Srinakharinwirot University, Bangkok 10110, Thailand; 4 School of Agriculture, Meiji University, Kawasaki, Kanagawa 214-8571, Japan; 5 Tsukuba Botanical Garden, National Museum of Nature and Science, Tsukuba, Ibaraki 305-0005, Japan

**Keywords:** *Dendrobium*, evolution, ITS, *matK*, Orchidaceae, phylogeny, systematics, taxonomy.

## Abstract

It is always difficult to construct coherent classification systems for plant lineages having diverse morphological characters. The genus *Dendrobium*, one of the largest genera in the Orchidaceae, includes ∼1100 species, and enormous morphological diversification has hindered the establishment of consistent classification systems covering all major groups of this genus. Given the particular importance of species in *Dendrobium* section *Dendrobium* and allied groups as floriculture and crude drug genetic resources, there is an urgent need to establish a stable classification system. To clarify phylogenetic relationships in *Dendrobium* section *Dendrobium* and allied groups, we analysed the macromolecular characters of the group*.* Phylogenetic analyses of 210 taxa of *Dendrobium* were conducted on DNA sequences of internal transcribed spacer (ITS) regions of 18S–26S nuclear ribosomal DNA and the maturase-coding gene (*matK*) located in an intron of the plastid gene *trnK* using maximum parsimony and Bayesian methods. The parsimony and Bayesian analyses revealed 13 distinct clades in the group comprising section *Dendrobium* and its allied groups. Results also showed paraphyly or polyphyly of sections *Amblyanthus*, *Aporum*, *Breviflores*, *Calcarifera*, *Crumenata*, *Dendrobium*, *Densiflora*, *Distichophyllae*, *Dolichocentrum*, *Holochrysa*, *Oxyglossum* and *Pedilonum*. On the other hand, the monophyly of section *Stachyobium* was well supported. It was found that many of the morphological characters that have been believed to reflect phylogenetic relationships are, in fact, the result of convergence. As such, many of the sections that have been recognized up to this point were found to not be monophyletic, so recircumscription of sections is required.

## Abstract (precis)

The genus *Dendrobium*, one of the largest genera in Orchidaceae, exhibits enormous vegetative diversification. Such a situation has hindered the establishment of consistent classification systems. To clarify phylogenetic relationships in *Dendrobium* section *Dendrobium* and allied groups, we performed molecular phylogenetic analyses of 210 taxa. The results showed that many sections are not monophyletic. Most of the morphological characters that have been believed to reflect phylogenetic relationships are, in fact, the results of convergence. Consequently, recircumscription of the infrageneric classification is required.

## Introduction

It is always difficult to construct coherent classification systems for plant lineages having diverse morphological characters. *Dendrobium* Sw. (Orchidaceae) represents such difficult groups and so far has been established in many alternative systems (e.g. Lindley 1830; Bentham and Hooker 1883; Kränzlin 1910; Schlechter 1912; Brieger 1981; Clements 2003; Wood 2006; Schuiteman 2011). This genus is one of the largest orchid genera, with around 1100 species (Wood 2006). The distribution range extends from Sri Lanka and India throughout tropical Asia and Oceania, north to Japan, east to Tahiti and south to New Zealand. Enormous diversification of the vegetative organs in accordance with habitat shifts and lack of accessory structures of the pollinarium, a cardinal character in orchid classification, has hindered establishment of consistent classification systems covering all major groups of this genus. Previous studies of systematics based on the morphological characteristics of the group were reviewed by Wood (2006). Given the limits to what can be understood of affinities using morphological characters, Yukawa *et al.* (1993) analysed the molecular phylogenetics of the subtribe Dendrobiinae (Lindley 1830), which includes the genus *Dendrobium* and putatively related genera *Cadetia*, *Diplocaulobium*, *Flickingeria*, *Epigeneium* and *Pseuderia* based on chloroplast DNA restriction site variation. This analysis resulted in presentation of the first probable phylogenetic relationship between members of this genus. Yukawa *et al.* (1993) demonstrated that *Dendrobium* is not monophyletic and comprises two major clades (Asian and Australasian clades: *sensu* Clements 2003). The Asian clade is predominantly diversified west of Weber's line, and the Australasian clade, containing genera *Cadetia*, *Diplocaulobium* and *Flickingeria*, is distributed mostly in Australasia and the Pacific Islands. Subsequent studies on representative members of subtribe Dendrobiinae (e.g. Yukawa *et al.* 1996, 1999, 2000; Yukawa 2001; Clements 2003, 2006; Schuiteman 2011) incorporated additional macromolecular markers and taxa in their analyses. In addition to providing further support for the above-mentioned phylogeny, these studies identified other infrageneric monophyletic groups.

*Dendrobium* section *Dendrobium* is one of the largest sections in the genus *Dendrobium*, comprising ∼60 species (Wood 2006) distributed across almost the entire geographical range of the genus, with the exception of Micronesia and Melanesia. A number of species are considered important as crude drug sources and are highly sought-after genetic resources with potential value in medicine (Takamiya *et al.* 2011, 2013). Yukawa *et al.* (1993) demonstrated that section *Dendrobium* is nested within the Asian clade. Wongsawad *et al.* (2001, 2005) analysed sequences for the maturase-coding gene (*matK*) located in the plastid genome and the internal transcribed spacer regions (ITS) of the nuclear ribosomal DNA of 78 Asian clade species including 35 of section *Dendrobium* (Wongsawad *et al.* 2001) and 93 Asian clade species including 42 members of section *Dendrobium* (Wongsawad *et al.* 2005). Based on these analyses, Wongsawad *et al.* demonstrated that section *Dendrobium* is not monophyletic; that its core clade includes species of sections *Breviflores*, *Densiflora*, *Holochrysa* and *Stuposa*; and that sections *Amblyanthus*, *Breviflores*, *Densiflora*, *Formosae*, *Holochrysa*, *Oxyglossum* and *Pedilonum* are not monophyletic. These relationships were confirmed by Xiang *et al.* (2013). However, given that these studies did not include several species of section *Dendrobium* and only included a small number of species in sections *Aporum*, *Calcarifera*, *Calyptrochilus*, *Crumenata*, *Distichophyllae*, *Oxyglossum*, *Pedilonum*, *Platycaulon*, *Stachyobium* and *Stuposa*, which are likely to be closely related to section *Dendrobium*, our understanding of the relationships between section *Dendrobium* and other groups within the Asian clade remains incomplete. In this study, we conducted comprehensive phylogenetic analyses of representative species in the Asian clade using the ITS and *matK* regions to clarify the relationships and the taxonomic position of section *Dendrobium*.

## Methods

### Plant materials

The samples for analysis consisted of 210 Asian clade species (214 samples), including 56 species belonging to section *Dendrobium*. As an outgroup, we chose 10 species of the Australasian clade, based on the results of Yukawa (2001). Plant materials were collected from the living collection of Tsukuba Botanical Garden, National Museum of Nature and Science. All voucher specimens were deposited at the Herbarium, National Museum of Nature and Science (TNS). Voucher information and GenBank accession numbers for all ITS and *matK* sequences used in this study are listed in Appendix 1. We adopted an infrageneric classification based on that proposed by Schlechter (1912) and modified by Wood (2006).

### DNA extraction and sequencing

Genomic DNA was isolated from fresh leaves or flowers by a DNeasy Plant Mini Kit (Qiagen, Hamburg, Germany) following the manufacturer's instructions, and was used as a template for PCR amplification. ITS1-5.8S-ITS2 regions were amplified and sequenced as described by Sun *et al.* (1994) and Hidayat *et al.* (2005). The amplification reactions included GC buffer I or II and LA Taq DNA polymerase (Takara Bio, Shiga, Japan). The PCR profile consisted of an initial 2-min premelt at 94 °C and 30 cycles of 50 s at 94 °C (denaturation), 1 min at 60 °C (annealing) and 30 s at 72 °C (extension), followed by a final 7-min extension at 72 °C. *matK* regions were amplified and sequenced as described by Hidayat *et al.* (2005). The amplification reactions included Ex Taq buffer and Ex Taq DNA polymerase (Takara Bio). The PCR profile consisted of an initial 5-min premelt at 94 °C and 30 cycles of 30 s at 94 °C (denaturation), 30 s at 53 °C (annealing) and 3 min at 72 °C (extension), followed by a final 7-min extension at 72 °C.

The PCR products were cleaned using a Montage PCR centrifugal filter device (Millipore, Billerica, MA, USA) and then sequenced in both forward and reverse directions using primers described by Hidayat *et al.* (2005). Sequences were obtained with an ABI PRISM 377 sequencer (Applied Biosystems, Foster City, CA, USA) following the manufacturer's instructions for auto-cycle sequencing reactions.

### Molecular phylogenetic analysis

Except for ITS and *matK* sequences of *Dendrobium ovatum* obtained from GenBank (accession ID for ITS: HM054721; *matK*: HM055325), all sequence data were obtained by our own analyses. Two hundred and twenty-four DNA sequences were aligned with ClustalW software. A phylogenetic analysis based on the maximum parsimony (MP) was performed using PAUP* version 4.0b10 (Swofford 2002) for three data sets: ITS, *matK* and a combination of the two. Gaps were treated as missing data. All characters were equally weighted and unordered (Fitch 1971). Each data set was analysed by a heuristic search method with tree bisection–reconnection branch swapping and the MULTREES option on 100 replications of random addition sequence with the stepwise addition option, and each replicate after 1 × 10^6^ rearrangements was assessed. Bootstrap support values were obtained from 1000 replicates using ‘fast’ stepwise addition. Although the fast stepwise addition analyses are expected to provide a lower support value than obtained when comprehensive branch-swapping is performed (DeBry and Olmstead 2000; Mort *et al.* 2000; Barkman *et al.* 2004), we used this option because bootstrap analyses with a full heuristic search method were not computationally feasible with a large data set. Bootstrap percentages (BPs) of 50–74 were defined as weak, 75–84 as moderate and 85–100 as strong, as in Chase *et al.* (2000) and Kim *et al.* (2010). The number of steps, consistency index (CI) and retention index (RI) were calculated with one of the most parsimonious trees in each analysis using the TREE SCORES command in PAUP*. To test congruence among data partitions, the incongruence length difference test (Farris *et al.* 1994, 1995), also designated the partition homogeneity test in PAUP*, was employed to measure character conflicts under a parsimony framework among data sets using 100 heuristic search replications.

The same data sets were analysed by Bayesian analysis using MrModeltest ver. 2.3 (Nylander 2004) to determine the sequence evolution model that best described the data. The GTR + I + G model was chosen for ITS, *matK* and the combined data by hierarchical likelihood ratio tests. The chosen model was used to perform a Bayesian analysis using the program MrBayes ver. 3.1.2 (Huelsenbeck and Ronquist 2001; Ronquist and Huelsenbeck 2003). For analysis, two simultaneous runs of four chains each were carried out with the Markov chain Monte Carlo algorithm for 8 000 000 generations, sampling every 100 generations. Data from the first 20 000 generations were discarded as the ‘burn-in’ period. The 50 % majority rule consensus phylogeny and posterior probability (PP) of nodes were calculated from the remaining samples. Clades with PP ≥95 were regarded as strongly supported (Martínez-Azorín *et al.* 2011).

### Ancestral state reconstruction of morphological characters

**Figure 1.**
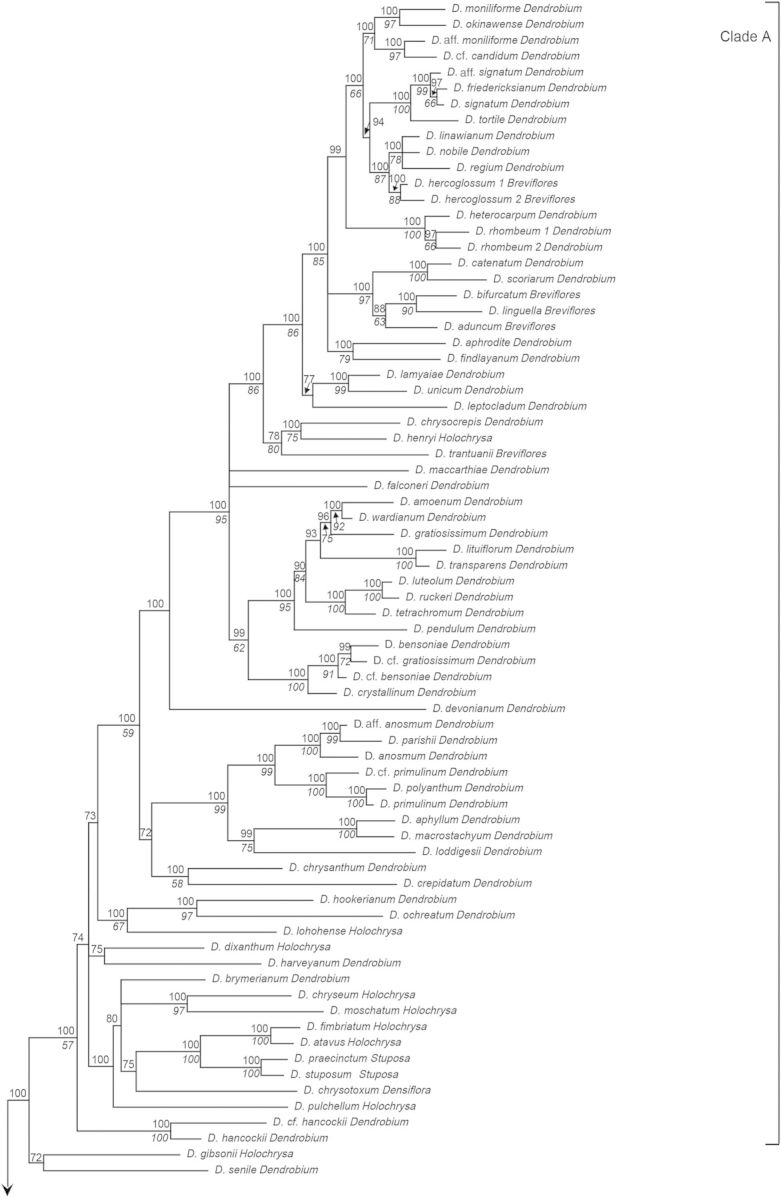

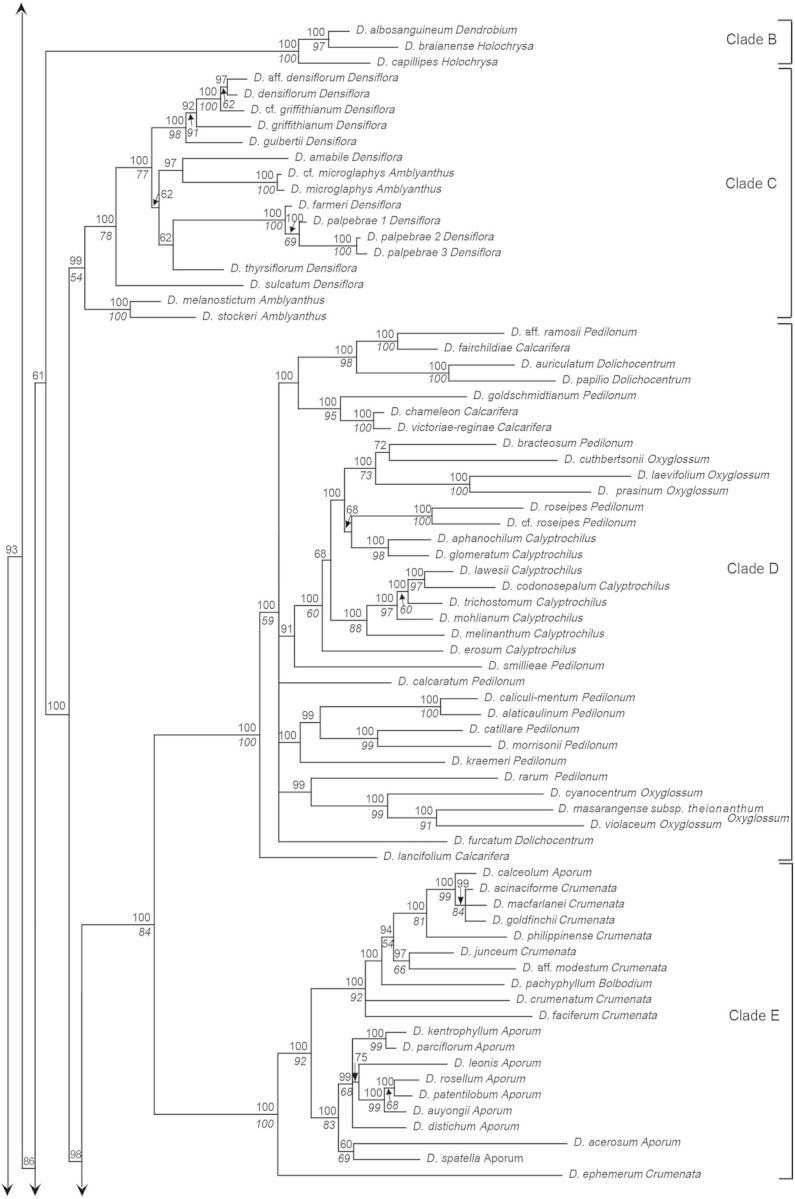

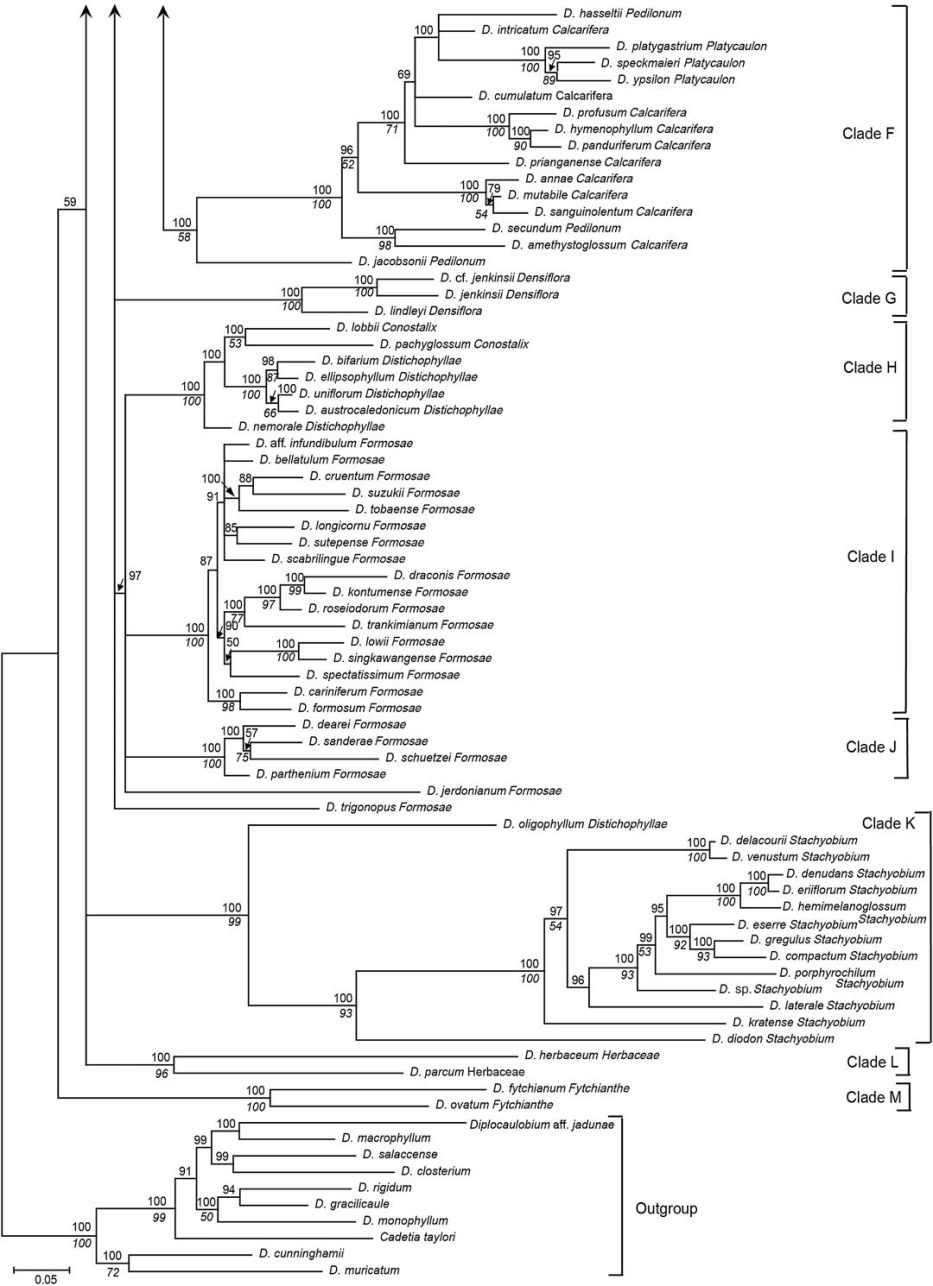
Consensus phylogram of 210 taxa of *Dendrobium* section *Dendrobium* and allied groups obtained from 96 596 Bayesian trees from the combined ITS and *matK* sequence data set. Values below and above branches indicate percentage bootstrap values from maximum parsimony analysis and Bayesian posterior probabilities, respectively.

Among various morphological characters, stem shape and leaf number were examined with ancestral state reconstruction analysis due to their complex pattern of character states even among closely related taxa. Character states for each taxon were obtained from the literature (Seidenfaden 1985; Wood 2006) and observation by the authors. A parsimony reconstruction was performed with the ‘Unordered’ model in Mesquite 2.75 (Maddison and Maddison 2011). The Bayesian tree based on the combined sequence data (ITS and *matK*) was used as the standard tree topology (see Fig. 1).

## Results

### Parsimony analyses of ITS, *matK* and combined sequences of the two

Table 1 shows statistics from ITS, *matK* and combined sequences. We compared topologies between strict consensus trees of ITS and *matK* data sets **[see Supporting Information]**. Visual inspection of the topologies between the two regions did not show significant incongruence, although an incongruence length difference test did not support congruence (*P* value = 0.01). Inconsistent topologies where bootstrap support is weak can be ignored as a soft incongruence (Johnson and Soltis 1998). Moreover, strongly supported clades by *matK* were generally strongly supported by ITS except for six inconsistent regions **[see** **Supporting Information****]**. Since analyses of combined data sets provide more resolution and internal support for relationships than individual data sets (Soltis *et al.* 1998), we combined ITS and *matK* data for further analysis.

**Table 1.** Characteristics of DNA datasets used in this study.

Characteristics	ITS	*matK*	Combined data
Number of taxa	224	224	224
Total number of characters	906	1630	2536
Number of constant characters	303	1092	1395
Number of parsimony uninformative characters	102	279	381
Number of parsimony informative characters	501	259	760
Tree length	5522	1092	6692
Consistency index (CI)	0.226	0.6016	0.2847
Retention index (RI)	0.715	0.7915	0.7198
Number of trees	140	4638	103
Bayesian model of evolution	GTR + I + G	GTR + I + G	GTR + I + G

In the majority of cases, BPs for trees based on the combined data set were higher than those obtained from analysis based on ITS or *matK* alone. For example, six clades supported by BP at <85 % in both genetic regions when based on either individual data set had BP >85 % in the combined case.

### Bayesian analyses of ITS, *matK* and combined sequences of the two

Comparing the Bayesian trees and strict consensus tree of the most parsimonious trees constructed from ITS and *matK* sequences, we found that in terms of the differences in results based on the sequence used, the clades of strict consensus trees that were strongly supported by bootstrap value in both ITS and *matK* trees were also strongly supported by the PP value **[see** **Supporting Information****]**. However, when we compared the strict consensus tree of the most parsimonious trees and the Bayesian trees with the ITS sequence, we found phylogenetic relationships in four parts where they were not strongly supported by PP value, even if they were by BP value. Specifically, these were the clade comprising *Dendrobium amoenum*, *D. gratiosissimum*, *D. wardianum*, *D. lituiflorum*, *D. transparens*, *D. luteolum*, *D. ruckeri* and *D. tetrachromum* (BP86, PP90); the clade comprising *D.* aff. *densiflorum*, *D. densiflorum*, *D.* cf. *griffithianum* and *D. griffithianum* (BP87, PP84); the clade comprising *D. farmeri* and *D. palpebrae* (BP87, PP75); and the clade comprising *D. speckmaieri* and *D. ypsilon* (BP89, PP92). That said, the PP values for these regions were still fairly high. Furthermore, as in the case for MP analysis, Bayesian analysis revealed that there are no inconsistencies between ITS and *matK* data sets, so the two regions were combined for subsequent analysis.

The combined tree obtained from a majority-rule consensus of 96 596 trees produced by two runs of the Markov chain Monte Carlo algorithm is presented in Fig. 1. With the exception of one clade consisting of *D.* aff. *densiflorum*, *D. densiflorum*, *D.* cf. *griffithianum* and *D. griffithianum* (BP91, PP92), all of the clades that were strongly supported in bootstrap tests based on MP analysis were also strongly supported by PP values in Bayesian analysis. In this study, major clades whose BP values from MP analysis were >50 % and those whose PP values from Bayesian analysis were >95 %—i.e. clades at least weakly supported by bootstrap values and strongly supported by PP values—were assigned letters A through M. Clade A comprises the majority of sections *Dendrobium* and *Holochrysa*, as well as sections *Breviflores* and *Stuposa*, and *Dendrobium chrysotoxum* in section *Densiflora*. Clade B comprises *Dendrobium albosanguineum* in section *Dendrobium* and *Dendrobium braianense* and *D. capillipes* in section *Holochrysa*. Clade C comprises the majority of section *Densiflora* and section *Amblyanthus*. Clade D consists of some members of sections *Pedilonum* and *Calcarifera* along with sections *Dolichocentrum*, *Oxyglossum* and *Calyptrochilus.* Clade E comprises sections *Aporum*, *Crumenata* and *Bolbodium.* Clade F comprises section *Platycaulon* and some members of sections *Pedilonum* and *Calcarifera*. Clade G comprises *D.* cf. *jenkinsii*, *D. jenkinsii* and *D. lindleyi* in section *Densiflora*. Clade H comprises section *Conostalix* and the majority of section *Distichophyllae*. Clade I comprises the majority of section *Formosae*. Clade J comprises *D. dearei*, *D. parthenium*, *D. sanderae* and *D. schuetzei* in section *Formosae*. Clade K comprises section *Stachyobium* and *Dendrobium oligophyllum* in section *Distichophyllae*. Clade L comprises section *Herbaceae*. Clade M comprises section *Fytchianthe*. No clear relationship to other clades was indicated for *Dendrobium gibsonii* in section *Holochrysa*, *D. senile* in section *Dendrobium*, or *D. jerdonianum* and *D. trigonopus* in section *Formosae*.

### Ancestral state reconstruction of morphological characters

**Figure 2.**
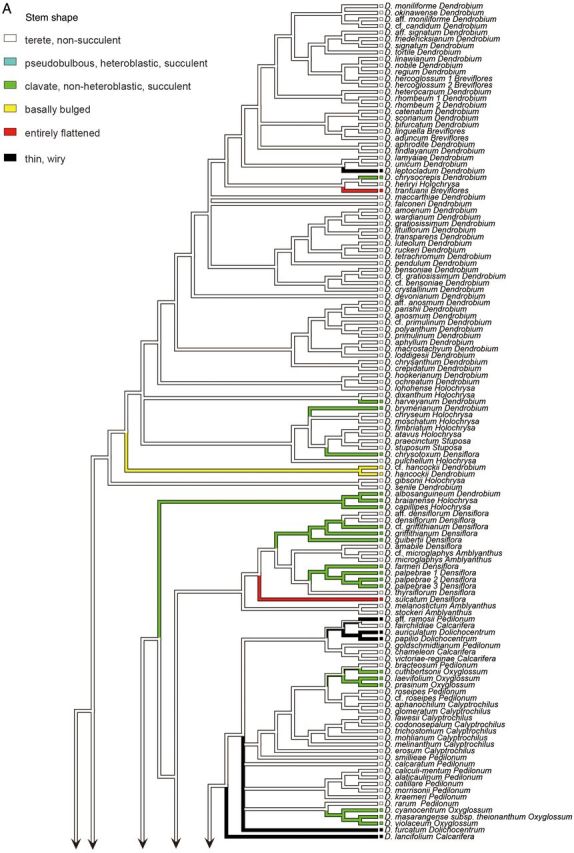

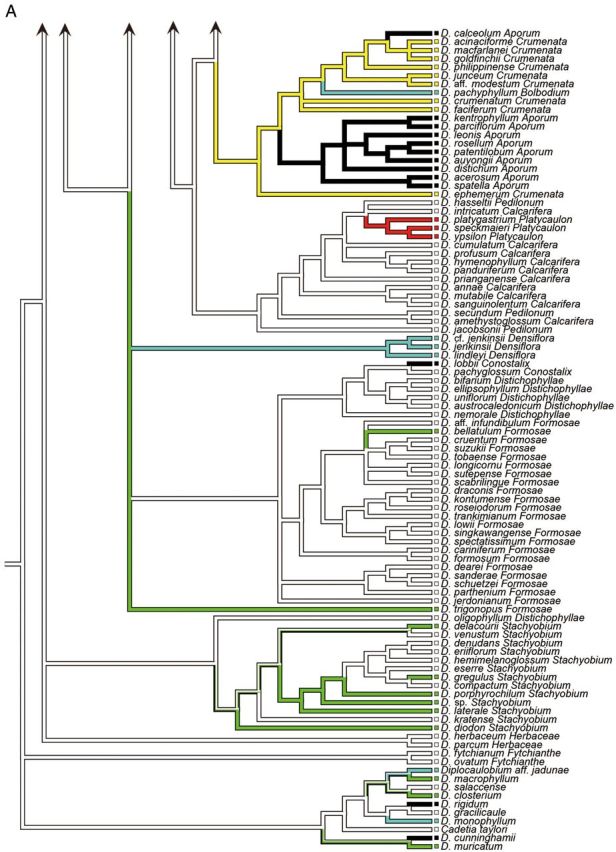

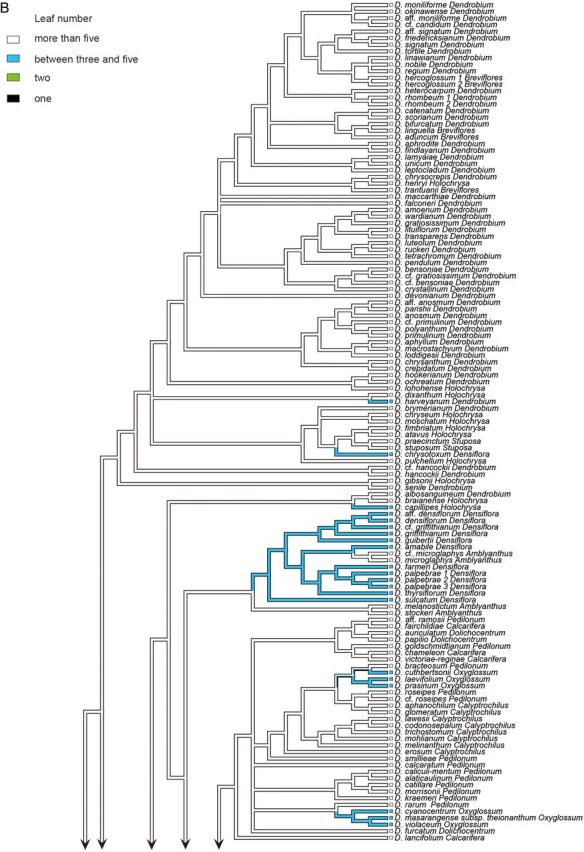

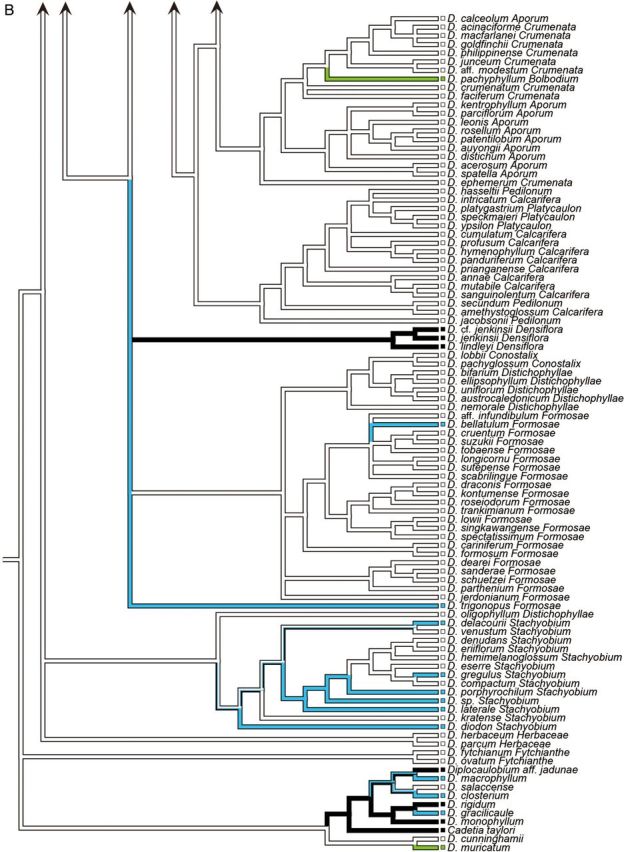
Ancestral state reconstruction of morphological characters ((A) stem shape; (B) leaf number) of 210 taxa of *Dendrobium* section *Dendrobium* and allied groups. All analyses were based on the parsimony reconstruction.

In the analysis of stem shape morphology, the terete, non-succulent stem was supported as the plesiomorphic state in the Asian clade (Fig. 2A). The other stem character states, namely, pseudobulbous, heteroblastic, succulent stem; clavate, non-heteroblastic, succulent stem; basally bulged stem; entirely flattened stem; and thin, wiry stem, were inferred to evolve twice, more than 14 times, twice, three times, and more than seven times, respectively. As for the evolution of leaf number, a character state bearing more than five leaves was suggested to be plesiomorphic in the Asian clade (Fig. 2B). The other character states of leaf number, namely, between three and five; two; and one, were inferred to evolve more than 10 times, once, and once, respectively.

## Discussion

### Phylogenetic relationships at the section level

Section *Dendrobium* is divided into three subclades. The first is the core clade, which includes the type species *Dendrobium moniliforme* (Clade A). The second is *D. albosanguineum*, included in Clade B, and the final subclade is *D. senile*, with a phylogenetic position that is unresolved. The possibility is suggested (PP72) that *D. senile* and *D. gibsonii* in section *Holochrysa* may form a clade. Furthermore, while there is a support of PP100 suggesting that these two species are sister groups to Clade A, due to the weak bootstrap support in MP analysis (BP < 50), they were not included in Clade A for the purposes of this study. In addition, Clade A also contains all species in section *Breviflores*, *D. chrysotoxum* in section *Densiflora*, all species in section *Stuposa*, and the majority of species in section *Holochrysa*, thus rendering section *Dendrobium* polyphyletic and paraphyletic. These results support the findings of Wongsawad *et al.* (2001, 2005) and Xiang *et al.* (2013).

Five species in section *Breviflores* (*Dendrobium aduncum*, *D. bifurcatum*, *D. hercoglossum*, *D. linguella* and *D. trantuanii*) are divided into three subclades within Clade A, making them a polyphyletic group. These results are consistent with the findings of Wongsawad *et al.* (2005) and Xiang *et al.* (2013). Two species in section *Stuposa* (*Dendrobium stuposum* and *D. praecinctum*) represent a monophyletic group within Clade A (BP100, PP100), supporting the findings of Wongsawad *et al.* (2005). Section *Holochrysa* was found to not be monophyletic, and its eight species (*Dendrobium atavus*, *D. chryseum*, *D. dixanthum*, *D. fimbriatum*, *D. henryi*, *D. lohohense*, *D. moschatum* and *D. pulchellum*) in Clade A are divided into six lineages. Further, a subclade comprising *D. braianense* and *D. capillipes* is placed within Clade B and the phylogenetic position of *D. gibsonii* is unresolved. These results are consistent with the analyses by Yukawa (2001), Wongsawad *et al.* (2001, 2005) and Xiang *et al.* (2013).

Section *Densiflora* was also found to not be monophyletic but rather to consist of three groups comprising the majority of Clade C, including the type species *D. densiflorum*, *D. chrysotoxum* belonging to Clade A, and Clade G, which is made up of *D. jenkinsii*, *D.* cf. *jenkinsii* and *D. lindleyi*. The analyses by Yukawa (2001), Wongsawad *et al.* (2001, 2005) and Xiang *et al.* (2013) also demonstrated the polyphyly of section *Densiflora*. Section *Amblyanthus* was found to nest within Clade C. Four species of section *Amblyanthus* were found to be divided into two subclades within Clade C, and were polyphyletic, supporting the results of Yukawa (2001) and Wongsawad *et al.* (2005).

Section *Pedilonum* was found to not be monophyletic, its members forming eight polyphyletic groups within Clade D and three polyphyletic groups within Clade F. The type species of section *Pedilonum*, *D. secundum*, was included in Clade F. Our results were consistent with analyses by Yukawa (2001), Wongsawad *et al.* (2001, 2005) and Clements (2003, 2006), which also revealed section *Pedilonum* to not be monophyletic.

Section *Calcarifera* represents another non-monophyletic group, which is divided into three subclades within Clade D and six subclades within Clade F. Consistent with our results, Clements (2003, 2006) also found that the section was not monophyletic and that its members formed two distinct groups. It is clear that the type species *Dendrobium pedicellatum*, which was not investigated in this study, belongs to Clade F because a morphologically related species, *Dendrobium mutabile*, is placed in this clade.

Clade D was shown to also include sections *Dolichocentrum*, *Oxyglossum* and *Calyptrochilus.* Section *Dolichocentrum* was found to be polyphyletic, with its three species splitting into a lineage represented by *Dendrobium furcatum* and a subclade (BP100, PP100) made up of *Dendrobium auriculatum* and *D. papilio*.

Section *Oxyglossum* was also found to not be monophyletic. Its six constituent species were divided into two groups, one comprising *Dendrobium cyanocentrum*, *D. masarangense* subsp*. theionanthum* and *D. violaceum* (BP99, PP100) and the other comprising *D. cuthbertsonii*, *D. laevifolium* and *D. prasinum*, along with *D. bracteosum*, a member of section *Pedilonum* (BP73, PP100). Consistent with our results, Clements (2003, 2006) also found that section *Oxyglossum* was not monophyletic and that its members split into two groups.

Our results suggested that section *Calyptrochilus* forms a polyphyletic group made up of three subclades: the first comprising *Dendrobium aphanochilum* and *D. glomeratum* (BP98, PP100); the second comprising *D. lawesii*, *D. codonosepalum*, *D. trichostomum*, *D. mohlianum* and *D. melinanthum* (BP88, PP100); and the third comprising *D. erosum*.

Section *Aporum* was found to be polyphyletic, nesting within Clade E along with sections *Crumenata* and *Bolbodium*. The members form two subclades: the first comprising nine species (BP83, PP100) and the second comprising *Dendrobium calceolum*. The monophyly of the first subclade was suggested by Yukawa (2001) and Wongsawad *et al.* (2001, 2005).

Section *Crumenata* was also found to nest within Clade E and to not be monophyletic. Its species are divided into two subclades: the first including section *Bolbodium* and parts of section *Aporum* (BP92, PP100) and the second representing the earliest diverging clade within Clade E, comprising *Dendrobium ephemerum*. Analyses by Yukawa (2001) and Clements (2003, 2006) also suggested the first of these subclades to be paraphyletic.

Section *Distichophyllae* was found to not be monophyletic, with its members splitting into a core clade (Clade H) containing the type species of the section, *Dendrobium uniflorum*, and the earliest diverging clade within Clade K represented by *D. oligophyllum*, which is likely a sister group to section *Stachyobium*. Further, in Clade H, two species of section *Conostalix*, *Dendrobium lobbii* and *D. pachyglossum*, were nested within members of section *Distichophyllae*. Section *Conostalix* is probably monophyletic (BP53, PP100).

Members of section *Formosae* were scattered into four lineages: Clade I, Clade J and two unplaced species, *D. jerdonianum* and *D. trigonopus.* Clade I, which contains the type species of the section, *D. formosum*, comprises only species of section *Formosae* (BP100, PP100). Clade J similarly comprises only species of section *Formosae* (BP100, PP100). While the possibility that these four groups represent a monophyletic lineage cannot be ruled out, it is likely that they are polyphyletic. Analyses by Wongsawad *et al.* (2005) and Clements (2006) also identified two clades within Section *Formosae* corresponding to Clades I and J.

There is a strong possibility that section *Stachyobium* forms a monophyletic group within Clade K (BP93, PP100). Consistent with our results, monophyly of section *Stachyobium* was also suggested by analyses by Yukawa (2001), Wongsawad *et al.* (2001, 2005), Clements (2006) and Xiang *et al.* (2013). Furthermore, it is evident that section *Stachyobium* is a sister group to *D. oligophyllum* in section *Distichophyllae* (BP99, PP100).

Section *Herbaceae* comprises only two species, *Dendrobium herbaceum* and *D. parcum*. We found that this section is clearly monophyletic because the two species constitute Clade L (BP96, PP100). Similarly, it is highly probable that section *Fytchianthe* represents a monophyletic lineage because *Dendrobium fytchianum* and *D. ovatum*, members of this section, constitute Clade M (BP100, PP100). While not strongly supported in statistical terms (BP < 50, PP59), Clade M may represent the earliest divergent clade within the Asian clade. Similarly supported by our results, analyses by Wongsawad *et al.* (2001, 2005) also indicate the possibility that section *Fytchianthe* is the earliest divergent clade within the Asian clade. Our analyses further indicate that sections *Herbaceae* and/or *Stachyobium* may represent the second-earliest divergent clade within the Asian clade after section *Fytchianthe*.

### Evolution of morphological characters

#### Vegetative stems

The vast majority of species belonging to Clades A, D, F, H, I, J, L and M, and several species in Clades C and K have terete, non-succulent vegetative stems. Results of ancestral state reconstruction analysis showed that this character state represents a plesiomorphy of the Asian clade (Fig. 2A).

Species with a pseudobulbous, heteroblastic, succulent stem in which from one to a few internodes on the upper portions of vegetative stems thicken to become pseudobulbs, account for all species in section *Bolbodium* and some members of sections *Densiflora*. Among species analysed in this study, the heteroblastic stem is exhibited by *Dendrobium pachyphyllum* (section *Bolbodium*) in Clade E, *D. lindleyi*, *D. jenkinsii* and *D.* cf. *jenkinsii* (all in section *Densiflora*) in Clade G. Results of ancestral state reconstruction analysis showed that this character state likely evolved twice in the Asian clade (Fig. 2A).

*Dendrobium brymerianum*, *D. chrysocrepis*, *D. harveyanum* (both in section *Dendrobium*) and *D. chrysotoxum* (section *Densiflora*) in Clade A, *D. albosanguineum* (section *Dendrobium*) and *D. braianense* and *D. capillipes* (both in section *Holochrysa*) in Clade B, *D.* cf. *griffithianum*, *D. farmeri*, *D. griffithianum*, *D. guibertii* and *D. palpebrae* (all in section *Densiflora*) in Clade C, all species of section *Oxyglossum* in Clade D, *D. bellatulum* (section *Formosae*) in Clade I, *D. delacourii*, *D. diodon*, *D. gregulus*, *D. porphyrochilum*, *D. laterale* and *Dendrobium* sp. (all in section *Stachyobium*) in Clade K and *D. trigonopus* (section *Formosae*), an unplaced taxon, have a clavate, non-heteroblastic, succulent stem. Results of ancestral state reconstruction analysis indicated that this character state evolved more than 14 times in the Asian clade (Fig. 2A).

All species of section *Crumenata*, and *Dendrobium hancockii* and *D*. cf. *hancockii* (both in section *Dendrobium*) exhibit a basally bulged stem characterized by particular thickening of several internodes positioned on lower portions of vegetative stems. Results of ancestral state reconstruction analysis showed that this character state represents an apomorphy, resulting from two independent evolutionary events within the Asian clade (Fig. 2A).

The stems of the majority of members of section *Calcarifera* have elliptical cross-sections, while the stems of all members of section *Platycaulon* are entirely flattened. A few species belonging to sections *Breviflores* and *Densiflora* also have flattened stems. Among species analysed in this study, in addition to species of section *Platycaulon* (Clade F), *D. trantuanii* (section *Breviflores*) in Clade A and *D. sulcatum* (section *Densiflora*) in Clade C also had flattened stems. Results of ancestral state reconstruction analysis showed that this character state evolved three times in the Asian clade (Fig. 2A).

A further apomorphy of the vegetative stem is thin, wiry stems, observed in all species of sections *Aporum* and *Dolichocentrum* and a few species of sections *Dendrobium*, *Calcarifera*, *Conostalix* and *Pedilonum*. Among species investigated in this study, in addition to species of sections *Aporum* and *Dolichocentrum*, thin and wiry stems were shared by *Dendrobium leptocladum* (section *Dendrobium*) in Clade A, *Dendrobium lancifolium* (section *Calcarifera*) and *Dendrobium* aff. *ramosii* (section *Pedilonum*) in Clade D and *D. lobbii* (section *Conostalix*) in Clade H. Results of ancestral state reconstruction analysis suggested that this character state evolved more than seven times in the Asian clade (Fig. 2A). Species belonging to section *Aporum* have succulent leaves, while other species with thin and wiry stems have fleshy roots. This suggests that, in species with thin and wiry stems, the components responsible for water storage have shifted from the stems to the leaves or roots.

#### Shoot architecture

The majority of species in the genus *Dendrobium* exhibit an architecture ubiquitously observed in perennial herbs in which vegetative shoots emerge in a repeated sympodial branching pattern from nodes on basal portions of the vegetative stem and produce roots also at the stem base. However, all species of section *Herbaceae* and a few species of sections *Dendrobium*, *Calcarifera*, *Dolichocentrum* and *Crumenata* produce vegetative shoots in a sympodial branching pattern from nodes on the upper parts of the stem, forming ramified stems, whereby roots do not grow from basal portions of new shoots. Among the species examined in this study, in addition to species of section *Herbaceae*, this architecture was observed in *Dendrobiuim falconeri* and *D. hancockii* (both in section *Dendrobium*) in Clade A; *D. chameleon* (section *Calcarifera*), *D. furcatum* (section *Dolichocentrum*) and *D. victoria-reginae* (section *Calcarifera*) in Clade D; and *D. junceum* (section *Crumenata*) in Clade E. This character state is not present in Clade M, which likely represents the earliest divergent clade within the Asian clade. In addition, given that this character state is only observed in a small number of species in a few lineages of both Asian and Australasian clades (Wood 2006), it is likely that ramified stems represent an apomorphy for the Asian clade, resulting from at least four distinct evolutionary events.

Although reproductive shoots are produced by axillary branching from vegetative shoots in most *Dendrobium* species, all species of sections *Stachyobium*, *Herbaceae* and *Fytchianthe* have terminal buds of vegetative shoots that develop into reproductive shoots. In this study, such a terminal inflorescence was observed in all species belonging to Clades M, L and K, which likely represent the earliest divergent clades within the Asian clade. Dressler (1981) proposed that terminal inflorescence is historically primary, being found in the most primitive orchid. Given that members of the genus *Epigeneium*, which represents the earliest divergence within subtribe Dendrobiinae, also exhibit a terminal inflorescence, terminal inflorescences may represent a plesiomorphy for the Asian clade.

#### Roots

Given that almost all Asian clade species have roots with a smooth, shiny white surface and that the majority of the sister Australasian clade species also have smooth roots, it appears that smooth roots represent a plesiomorphy for the Asian clade. In contrast, a number of species belonging to Clade F (*Dendrobium annae*, *D. cumulatum*, *D. hymenophyllum*, *D. intricatum*, *D. mutabile*, *D. panduriferum*, *D. profusum* and *D. sanguinolentum* in section *Calcarifera* and *D. platygastrium*, *D. speckmaieri* and *D. ypsilon* in section *Platycaulon*) have verrucose roots. All members of section *Brevisaccata* in the Australasian clade and some species of the genus *Epigeneium* also exhibit this feature. Such roots certainly evolved three times in the subtribe Dendrobiinae. However, this rough surface feature has no known function, except for increasing the absorptive surface of the root (Wood 2006).

#### Leaves

While almost all species belonging to the Asian clade have conduplicate leaves with both adaxial and abaxial surfaces, all species in section *Aporum* and some species in section *Crumenata* have laterally flattened or terete leaves in which the adaxial surface has been reduced, leaving a unifacial leaf consisting only of the abaxial surface. This feature is accompanied by thickening of the mesophyll, and these character states represent a xerophytic adaptation. In fact, the species with unifacial leaves generally grow as epiphytes in dry habitats of tropical Asia (Yukawa and Uehara 1996). Among the species analysed in this study, all species in section *Aporum* and section *Crumenata* species *Dendrobium acinaciforme*, *D. macfarlanei*, *D. goldfinchii*, *D. philippinense*, *D. junceum* and *D.* aff. *modestum* in Clade E exhibit a unifacial leaf. Since evolution of a unifacial leaf occurred only in Clade E in the Asian clade, it represents an apomorphy resulting from one or a few evolutionary events.

Members of the genus *Dendrobium* exhibit two shedding patterns: a deciduous pattern, in which leaf life-span is shorter than 1 year and stems lose all leaves temporarily, and an evergreen pattern, in which leaf life-span is longer than 1 year and stems retain their leaves at all times. While the vast majority of species are evergreen, all species in sections *Herbaceae*, *Fytchianthe* and *Stachyobium* and some members in sections *Dendrobium* and *Holochrysa* are deciduous. Among species investigated in this study, in addition to species of sections *Herbaceae*, *Fytchianthe* and *Stachyobium*, section *Dendrobium* species *D. aphyllum*, *D. crystallinum*, *D. pendulum*, *D. wardianum*, *D. amoenum*, *D. anosmum*, *D. bensoniae*, *D. crepidatum*, *D. devonianum*, *D. gratiosissimum*, *D. lituiflorum*, *D. parishii*, *D. primulinum*, *D. transparens*, *D. unicum* and *D. dixanthum* (section *Holochrysa*) in Clade A, along with *D. albosanguineum* (section *Dendrobium*), *D. capillipes* (section *Holochrysa*) in Clade B and *D. senile* (section *Dendrobium*), with unresolved phylogenetic position, are all deciduous. Furthermore, while members of the Australasian clade as well as *Epigeneium* and *Bulbophyllum*, allied genera to *Dendrobium*, have evergreen leaves, given that species of sections *Fytchianthe*, *Herbaceae* and *Stachyobium*, which likely are the earliest divergent clades within the Asian clade, are deciduous, deciduousness may represent a plesiomorphy for the Asian clade.

Results of ancestral state reconstruction analysis showed that a stem with more than five leaves represents a plesiomorphy of the Asian clade and that reduction of leaf number likely evolved more than 12 times (Fig. 2B). As shown in Fig. 2A and B, the species with less than six leaves on each stem usually exhibit pseudobulbous, heteroblastic, succulent or clavate, non-heteroblastic, succulent stems except for *D.* aff. *densiflorum*, *D. amabile*, *D. densiflorum*, *D. sulcatum* and *D. thyrsiflorum* (all in section *Densiflora*) in Clade C. Furthermore, leaves of these species are either succulent or deciduous. Yukawa and Uehara (1996) and Wood (2006) suggested that these combinations of character states show adaptation to a xeric environment.

Schlechter (1912), in his infrageneric classification system of *Dendrobium*, emphasized the presence or absence of leaf sheaths as a cardinal diagnostic character at the subgenus level. In species with the above-mentioned succulent stems producing a small number of leaves in the upper part, leaf sheaths do not develop. However, in species that produce non-succulent stems, leaf sheaths do not develop in the uppermost leaves, but do develop in lower leaves. Consequently, reduction of the leaf sheath in the uppermost leaves is a common character state both in the succulent and non-succulent stem species, and it is not appropriate to use the presence or absence of leaf sheaths as a character for classification at least in the Asian clade.

#### Anatomical characters of vegetative organs

Yukawa and Uehara (1996) demonstrated that modifications in size and number of parenchymatous cells of vegetative stems substantially contributed to vegetative diversification in *Dendrobium*. This observation implies that a simple structural adjustment can result in a major modification of growth habit in this group. They also found that several anatomical characters associated with xeromorphy, such as a thick outer wall of epidermal cells on the stem surface and a thick sclerenchymatous cap on vascular bundles in the stem evolved in members of sections *Aporum* and *Crumenata*. In other words, these character states are likely to have evolved in the common ancestor of Clade E. Acquisition of xeromorphic anatomical characters in this clade may have facilitated evolution of unique vegetative characters suited to dry environments such as a succulent, laterally flattened leaf and a wiry or basally bulged stem.

Namba and Lin (1981*a*, *b*), Singh (1986) and Morris *et al.* (1996) investigated the anatomical characters of roots of *Dendrobium.* Morris *et al.* demonstrated that root characteristics including the number of layers in the velamina are not useful characters to use in determining sectional relationships.

#### Reproductive organs

All species of section *Densiflora* and most members of section *Dendrobium* have velvety lips with many papillae on the surface. Among species analysed in this study, all Clade A species with the exception of *D. unicum* and *D. lamyaiae* in section *Dendrobium*, all Clade B, C and G species, and *D. gibsonii* exhibit this character state. When *Dendrobium* flowers are stained with neutral red, which is taken up by osmophores, or odour-producing cells (Stern *et al.* 1986), the papillae of the above-listed species are strongly stained (Yukawa 1993). Consequently, it is likely that the papillae on the lip surface accumulate substances responsible for fragrance (Müller 1935). Observation of the near-ultraviolet (near-UV) reflectance of the flowers using near-UV reflectance photography reveals that the parts with papillae absorb near-UV, while other areas of the perianth lobes reflect near-UV, resulting in distinct high-contrast patterns (Yukawa 1993; Indsto and Weston 2000). Given that this combination of characteristics is typical of bee-pollinated flowers, we demonstrate that the dense packing of papillae on lip surfaces represents a character state that evolved in adaptation to bee pollination. The only *Dendrobium* species producing lips with dense papillae for which pollinators have been observed is *D. anosmum*, which two species of *Apis* are known to pollinate (Burkill 1919). Since lip surfaces with dense papillae are not observed in species of the Australasian clade or in Clades K, L and M, which likely represent the earliest divergent clades within the Asian clade, this character state was probably acquired after the divergence of the Asian clade. However, it is unclear how many evolutionary events have resulted in the character state, because phylogenetic relationships of Clades A, B, C and G have not been resolved.

Meanwhile, specialization has occurred in terms of flowering behaviour. Reproductive shoots of *Dendrobium* generally branch sympodially from vegetative shoots and disappear immediately after flowering. However, in all species in Clade E, namely, members of sections *Aporum*, *Bolbodium* and *Crumenata*, the reproductive shoots remain alive even after flowering and continue to flower repeatedly. There is a high probability that the repeated flowering behaviour was acquired by a common ancestor of Clade E. Further, all species of section *Bolbodium* and some species of sections *Crumenata* and *Aporum* in this clade exhibit gregarious flowering in which, after the flower has differentiated, its growth is suspended at the bud stage and all flowers begin to grow and then bloom at once when the temperature decreases to a certain level or when the difference between daytime and night-time temperatures narrows to a certain point (Seifriz 1923; Coster 1925; Gerlach 1992). This behaviour is observed, for example, in *D. pachyphyllum* (section *Bolbodium*), *D. crumenatum* (section *Crumenata*), *D. ephemerum* (section *Crumenata*), *D. acerosum* (section *Aporum*) and *D. spatella* (section *Aporum*). It is possible that acquisition of repeated flowering in the common ancestor of Clade E provided further character evolution that increased plant attractiveness to pollinators, because it enabled an individual plant to have numerous buds at once. Acquisition of repeated flowering thus may trigger the evolution of gregarious flowering.

### Reappraisal of infrageneric classification

There is no single criterion, but rather, several options for deciding what level of monophyletic unit warrants designation as a genus. In order to fulfil the phylogenetic consistency and conservation of a widely accepted concept of the genus *Dendrobium*, Yukawa *et al.* (1993, 1996, 1999, 2000), Yukawa (2001), Burke *et al.* (2008), Adams (2011) and Schuiteman (2011) have recommended expanding the genus *Dendrobium* to include the genera *Cadetia*, *Diplocaulobium* and *Flickingeria.* In contrast, Clements (2003, 2006) subdivided *Dendrobium* and established many new genera to recover the monophyly of the genus. The advantages and disadvantages of these two approaches were discussed by Yukawa *et al.* (1999) and Schuiteman and Adams (2011), who concluded that the former approach has greater merit. Here, we follow the former approach and examine the infrageneric classification system of *Dendrobium* proposed by Wood (2006).

We demonstrated that a number of sections used by Wood (2006) are not monophyletic. In order to restore the monophyly of these groups, a revised system at the section level is necessary. Clades A through M defined in this study were all strongly supported, indicating that they are stable units whose monophyly will continue to be supported even if the sampling intensity and gene regions analysed were increased. Furthermore, given that considerable numbers of clades roughly correspond to traditionally used sections, it would seem reasonable to assign, for the most part, Clades A through M to the section level.

We found that in Clade A, section *Dendrobium* is a paraphyletic group comprising all species in sections *Breviflores* and *Stuposa*, most species in section *Holochrysa* and *D. chrysotoxum* in section *Densiflora*. If we recognize these heterogeneous elements at the section level, section *Dendrobium* also must be subdivided. As sections *Breviflores*, *Stuposa*, *Holochrysa* and *D. chrysotoxum* in *Densiflora* do not deviate from a broad concept of section *Dendrobium*, it would be appropriate to redefine section *Dendrobium* as the range of Clade A and to include sections *Breviflores* and *Stuposa*, and most species in section *Holochrysa*, and *D. chrysotoxum* of section *Densiflora* in section *Dendrobium*. Schuiteman (2011) also suggested that section *Breviflores* is polyphyletic and should be included in section *Dendrobium*. Further, Xiang *et al.* (2013) proposed to subsume these three sections and *D. chrysotoxum* into section *Dendrobium*. Our analyses indicated that *D. gibsonii* (section *Holochrysa*) and *D. senile* (section *Dendrobium*) may form a monophyletic group with Clade A (BP < 50, PP100). At present, it is appropriate to include these two species in section *Dendrobium*.

Clade B can be characterized by the following combination of characters: clavate, non-heteroblastic, succulent stems; long flowering stems produced from the upper part of vegetative stems; lip with numerous papillae on the adaxial surface; tapered anther cap; and leaf sheath with brownish margins. Among these characters, leaf sheath with brownish margins represents synapomorphy of this clade. While Clade B itself is strongly supported (BP100, PP100), its relationship to other clades remains to be resolved. Therefore, it would seem appropriate to treat Clade B as an independent section. Clade B is comprised of members of sections *Dendrobium* and *Holochrysa*, both of whose type species belong to Clade A. Given that no name corresponding to the three species constituting Clade B currently exists, a new section name will have to be assigned.

Clade C comprises sections *Densiflora* and *Amblyanthus*, both of which are not monophyletic in the clade. *Dendrobium microglaphys* of section *Amblyanthus* occupies a nested position among members of section *Densiflora* and lacks scales outside the perianth lobes that characterize section *Amblyanthus*; instead, it has reduced leaf sheaths and pendulous flowering stems, both of which are diagnostic characters of section *Densiflora*. Consequently, it is reasonable to transfer *D. microglaphys* to section *Densiflora*. Meanwhile, the members of the basal subclade of Clade C, which includes *Dendrobium melanostictum*, the type species of section *Amblyanthus*, share features such as scales outside the perianth lobes, short racemes, and a peculiar scaly covering of the flowers and a backward-pointing lip appendage. Therefore, this subclade corresponds to section *Amblyanthus* and the rest of Clade C can be defined as section *Densiflora*.

Clade D comprises sections *Pedilonum*, *Calcarifera*, *Oxyglossum*, *Calyptrochilus* and *Dolichocentrum.* Since sections *Pedilonum*, *Calcarifera*, *Oxyglossum* and *Dolichocentrum* are not monophyletic, redefinition of these sections is necessary. If we conserve these sections, establishment of a large number of new sections is required. To avoid this complexity, we suggest designating Clade D as a single section. For this purpose, the section names *Pedilonum* and *Calcarifera* are not appropriate since their type species belong to Clade F. Therefore, the oldest available section name for Clade D is *Calyptrochilus*. An elongated mentum characterizes all species in this clade, while this character also defines Clade F and several species in other clades.

Clade E comprises sections *Aporum*, *Crumenata* and *Bolbodium*, the first two of which were found to not be monophyletic. As in the case of Clade D, conserving these two section names requires the establishment of multiple new sections. Thus, it is better to treat Clade E as a single section. The oldest available name corresponding to this clade is section *Aporum*. Clade E species have the following synapomorphies: a thick outer wall of epidermal cells on the stem surface and a thick sclerenchymatous cap on vascular bundles in the stem (Yukawa and Uehara 1996), and repeated flowering behaviour from the same reproductive shoot. Schuiteman (2011) also suggested that sections *Aporum*, *Crumenata* and *Bolbodium* should be treated as a single section.

Clade F comprises sections *Pedilonum*, *Calcarifera* and *Platycaulon*, the first two of which were found to not be monophyletic. Again, to avoid complexity by naming many new sections, it is reasonable to designate Clade F as a single section. The oldest available name corresponding to this clade is section *Pedilonum*. A shared character for Clade F is an elongated mentum, but this character also appeared in other clades, as mentioned previously.

The three species constituting Clade G have previously been treated as section *Densiflora*. Since we redefine section *Densiflora* as a subclade in Clade C where the type species is included, this section name cannot be used in Clade G. While the monophyly of this clade is strongly supported (BP100, PP100), its relationship to other clades remains to be resolved. Consequently, it is appropriate to deal with Clade G as an independent section. Given that no name corresponding to the three species constituting Clade G currently exists, a new section name will have to be assigned. Xiang *et al.* (2013) also suggested a separate status for a clade comprising *D. jenkinsii* and *D. lindleyi*. Members of this clade share the following combination of characters: pseudobulbous, heteroblastic, succulent stem, single leaf on the stem, and a few long flowering stems from the apical part of the vegetative stem. Among these characters, a single leaf represents the synapomorphy of this clade.

Clade H consists of members of sections *Distichophyllae* and *Conostalix*. As the latter section is nested within the first, the first section becomes paraphyletic. Except for thinner vegetative stems in section *Conostalix*, there is no obvious character to distinguish the two sections. Therefore, we deem it appropriate to redefine section *Distichophyllae* as the range of Clade H.

Clades I and J as well as unplaced species *D. jerdonianum* and *D. trigonopus* compose section *Formosae*. In addition to the definite monophyletic status of Clades I and J, it cannot be ruled out that some or all clades of this section along with section *Distichophyllae* form a monophyletic group. Since further analyses may resolve the ambiguous relationships of these clades, we suspend taxonomic treatment of section *Formosae* for the moment.

Clade K comprises section *Stachyobium* and *D. oligophyllum* of section *Distichophyllae*, which is the earliest divergent lineage in this clade. Re-examination of *D. oligophyllum* showed that the morphological characters of this species are consistent with the definition of section *Stachyobium* except for the long life-span of the leaf in this species. We thus transfer *D. oligophyllum* to section *Stachyobium*, and by this treatment, Clade K coincides with section *Stachyobium*. While the species of section *Stachyobium* share terminal inflorescences, we did not identify any synapomorphies of this section.

Clades L and M correspond to sections *Herbaceae* and *Fytchianthe*, respectively, endorsing the validity of the current definition of these sections. The species of section *Herbaceae* are characterized by ramified stems, terminal inflorescences and deciduous leaves. The species of section *Fytchianthe* are characterized by terminal inflorescences and deciduous leaves.

The scope of this study did not include the Australasian clade, the other major clade in genus *Dendrobium*. While a number of molecular phylogenetic studies have been conducted for this clade as well (Y. T. unpubl. res.; Yukawa *et al.* 1993, 1996, 2000; Yukawa 2001; Clements 2003, 2006; Burke *et al.* 2008), no attempt has been made to comprehensively analyse this clade as a whole. A consistent infrageneric classification of this genus should be proposed after combining the results of a phylogenetic analysis of both Asian and Australasian clades.

## Sources of Funding

This work was partially supported by a Grant-in-Aid for Scientific Research (24370040 to T.Y.) from the Ministry of Education, Culture, Sports, Science and Technology, Japan.

## Contributions by the Authors

T.Y., T.T., T.H., S.K. and H.I. conceived and designed the study. T.Y., T.T., P.W., A.S., T.N., S.S., S.K. and N.S. performed sequence and phylogenetic analyses. T.Y. and T.T. primarily wrote the manuscript with contributions from all the authors.

## Conflicts of Interest Statement

None declared.

## Supporting Information

The following Supporting Information is available in the online version of this article –

**Figure S1.** Consensus phylogram obtained from 100 754 Bayesian trees based on ITS sequences. Values below and above branches indicate percentage bootstrap values from maximum parsimony analysis and Bayesian posterior probabilities, respectively.

**Figure S2.** Consensus phylogram obtained from 119 935 Bayesian trees based on *matK* sequences. Values below and above branches indicate percentage bootstrap values from maximum parsimony analysis and Bayesian posterior probabilities, respectively.

Additional Information

## Appendix

**Appendix 1.** List of *Dendrobium* taxa and GenBank IDs.

Species	Section	Voucher	ITS	*matK*
*D. acerosum*	*Aporum*	Yukawa04-32	AB847639	AB847678
*D. acinaciforme*	*Crumenata*	TBG120483	AB847640	AB847679
*D. aduncum*	*Breviflores*	TBG134842	AB593484	AB847680
*D.* aff. *anosmum*	*Dendrobium*	Ting H19	AB593490	AB847686
*D.* aff. *densiflorum*	*Densiflora*	TBG159449	AB593485	AB847681
*D.* aff. *infundibulum*	*Formosae*	TBG128905	AB593486	AB847682
*D.* aff. *modestum*	*Crumenata*	TBG119268	AB847642	AB847683
*D.* aff. *moniliforme*	*Dendrobium*	TBG124453	AB593488	AB847684
*D.* aff. *ramosii*	*Pedilonum*	HBG4335	AB593492	AB847687
*D.* aff. *signatum*	*Dendrobium*	TBG118279	AB593489	AB847685
*D. alaticaulinum*	*Pedilonum*	TBG135586	AB593493	AB847688
*D. albosanguineum*	*Dendrobium*	TBG122507	AB593494	AB847689
*D. amabile*	*Densiflora*	TBG111421	AB593495	AB847690
*D. amethystoglossum*	*Calcarifera*	TBG120501	AB593496	AB847691
*D. amoenum*	*Dendrobium*	TBG159411	AB593497	AB847692
*D. annae*	*Calcarifera*	TBG111422	AB593498	AB847693
*D. anosmum*	*Dendrobium*	TBG118511	AB593499	AB847694
*D. aphanochilum*	*Calyptrochilus*	TBG142252	AB593500	AB847695
*D. aphrodite*	*Dendrobium*	TBG122797	AB593501	AB847696
*D. aphyllum*	*Dendrobium*	TBG122508	AB593539	AB847736
*D. atavus*	*Holochrysa*	TBG159413	AB593502	AB847697
*D. auriculatum*	*Dolichocentrum*	TBG120492	AB593503	AB847698
*D. austrocaledonicum*	*Distichophyllae*	TBG130055	AB593504	AB847699
*D. auyongii*	*Aporum*	TBG123341	AB847643	AB847700
*D. bellatulum*	*Formosae*	TBG133256	AB847644	AB847701
*D. bensoniae*	*Dendrobium*	TBG128899	AB593505	AB847702
*D. bifarium*	*Distichophyllae*	TBG159414	AB593506	AB847703
*D. bifurcatum*	*Breviflores*	TBG157293	AB593507	AB847704
*D. bracteosum*	*Pedilonum*	TBG102743	AB593509	AB847705
*D. braianense*	*Holochrysa*	TBG159424	AB593510	AB847706
*D. brymerianum*	*Dendrobium*	TBG118826	AB593511	AB847707
*D. calcaratum*	*Pedilonum*	TBG125164	AB593512	AB847708
*D. calceolum*	*Aporum*	TBG122494	AB593513	AB847709
*D. caliculi-mentum*	*Pedilonum*	TBG118610	AB593514	AB847710
*D. capillipes*	*Holochrysa*	TBG128878	AB593515	AB847711
*D. cariniferum*	*Formosae*	TBG128900	AB847645	AB847712
*D. catenatum*	*Dendrobium*	TBG159450	AB593517	AB847713
*D. catillare*	*Pedilonum*	TBG124769	AB847646	AB847714
*D.* cf. *bensoniae*	*Dendrobium*	036193-B	AB847647	AB847715
*D.* cf. *candidum*	*Dendrobium*	29746	AB593519	AB847716
*D.* cf. *gratiosissimum*	*Dendrobium*	TBG126713	AB593522	AB847719
*D.* cf. *griffithianum*	*Densiflora*	TBG128876	AB593523	AB847720
*D.* cf. *hancockii*	*Dendrobium*	TBG142251	AB593524	AB847721
*D.* cf. *jenkinsii*	*Densiflora*	TBG129904	AB593525	AB847722
*D.* cf. *microglaphys*	*Amblyanthus*	TBG116027	AB593526	AB847723
*D.* cf. *primulinum*	*Dendrobium*	TBG118293	AB593521	AB847718
*D.* cf. *roseipes*	*Pedilonum*	TBG159460	AB847648	AB847717
*D. chameleon*	*Calcarifera*	TBG135506	AB593527	AB847724
*D. chrysanthum*	*Dendrobium*	TBG124319	AB593529	AB847725
*D. chryseum*	*Holochrysa*	TBG127382	AB593530	AB847726
*D. chrysocrepis*	*Dendrobium*	TBG128879	AB593531	AB847727
*D. chrysotoxum*	*Densiflora*	TBG118321	AB593533	AB847728
*D. codonosepalum*	*Calyptrochilus*	TBG137050	AB847649	AB847729
*D. compactum*	*Stachyobium*	TBG133060	AB847650	AB847730
*D. crepidatum*	*Dendrobium*	TBG128871	AB593534	AB847731
*D. cruentum*	*Formosae*	TBG134572	AB593536	AB847733
*D. crumenatum*	*Crumenata*	TBG115833	AB593537	AB847734
*D. crystallinum*	*Dendrobium*	TNS8502972	AB593538	AB847735
*D. cumulatum*	*Calcarifera*	TBG159418	AB593541	AB847737
*D. cuthbertsonii*	*Oxyglossum*	TBG116108	AB593542	AB847738
*D. cyanocentrum*	*Oxyglossum*	TBG159420	AB593543	AB847739
*D. dearei*	*Formosae*	TBG144587	AB847651	AB847740
*D. delacourii*	*Stachyobium*	TBG100237	AB593545	AB847741
*D. densiflorum*	*Densiflora*	TBG159421	AB593546	AB847742
*D. denudans*	*Stachyobium*	TBG132760	AB593547	AB847743
*D. devonianum*	*Dendrobium*	TBG124383	AB593548	AB847744
*D. diodon*	*Stachyobium*	TBG116089	AB593550	AB847745
*D. distichum*	*Aporum*	TBG137051	AB593551	AB847746
*D. dixanthum*	*Holochrysa*	TBG128877	AB593552	AB847747
*D. draconis*	*Formosae*	TBG118276	AB593553	AB847748
*D. ellipsophyllum*	*Distichophyllae*	TBG126635	AB593554	AB847749
*D. ephemerum*	*Crumenata*	TBG159426	AB593555	AB847750
*D. eriiflorum*	*Stachyobium*	TBG140557	AB593556	AB847751
*D. erosum*	*Calyptrochilus*	TBG159429	AB593557	AB847752
*D. eserre*	*Stachyobium*	TBG133168	AB593558	AB847753
*D. faciferum*	*Crumenata*	YukawaDNA2134	AB847652	AB847754
*D. fairchildiae*	*Calcarifera*	TBG120506	AB593559	AB847755
*D. falconeri*	*Dendrobium*	TBG128903	AB593560	AB847756
*D. farmeri*	*Densiflora*	TBG159430	AB593561	AB847757
*D. fimbriatum*	*Holochrysa*	84386	AB593562	AB847758
*D. findlayanum*	*Dendrobium*	TBG128904	AB593563	AB847759
*D. formosum*	*Formosae*	TBG128902	AB593564	AB847760
*D. friedericksianum*	*Dendrobium*	TBG124322	AB593565	AB847761
*D. furcatum*	*Dolichocentrum*	TBG116070	AB593566	AB847762
*D. fytchianum*	*Fytchianthe*	TBG128868	AB593567	AB847763
*D. gibsonii*	*Holochrysa*	TBG129876	AB593568	AB847764
*D. glomeratum*	*Calyptrochilus*	TBG138022	AB593535	AB847732
*D. goldfinchii*	*Crumenata*	Yukawa 97-2008	AB593569	AB847765
*D. goldschmidtianum*	*Pedilonum*	TBG159434	AB593570	AB847766
*D. gratiosissimum*	*Dendrobium*	TBG126651	AB593571	AB847767
*D. gregulus*	*Stachyobium*	TBG132862	AB593572	AB847768
*D. griffithianum*	*Densiflora*	TBG138067	AB593573	AB847769
*D. guibertii*	*Densiflora*	TBG132542	AB593574	AB847770
*D. hancockii*	*Dendrobium*	TBG122506	AB593575	AB847771
*D. harveyanum*	*Dendrobium*	TBG133184	AB593576	AB847772
*D. hasseltii*	*Pedilonum*	TBG136022	AB593577	AB847773
*D. hemimelanoglossum*	*Stachyobium*	TBG133257	AB593578	AB847774
*D. henryi*	*Holochrysa*	TBG127415	AB593579	AB847775
*D. herbaceum*	*Herbaceae*	TBG156719	AB847654	AB847776
*D. hercoglossum_*1	*Breviflores*	TBG118850	AB593580	AB847777
*D. hercoglossum_*2	*Breviflores*	TBG124432	AB593581	AB847778
*D. heterocarpum*	*Dendrobium*	TBG116506	AB593582	AB847779
*D. hookerianum*	*Dendrobium*	TBG133007	AB593584	AB847780
*D. hymenophyllum*	*Calcarifera*	TBG140599	AB847655	AB847781
*D. intricatum*	*Calcarifera*	TBG137294	AB593586	AB847782
*D. jacobsonii*	*Pedilonum*	TBG133080	AB593587	AB847783
*D. jenkinsii*	*Densiflora*	TBG129880	AB593589	AB847784
*D. jerdonianum*	*Formosae*	TBG156717	AB847656	AB847785
*D. junceum*	*Crumenata*	TBG119079	AB593590	AB847786
*D. kentrophyllum*	*Aporum*	TBG102615	AB593591	AB847787
*D. kontumense*	*Formosae*	TBG126642	AB593592	AB847788
*D. kraemeri*	*Pedilonum*	TBG123234	AB847657	AB847789
*D. kratense*	*Stachyobium*	TBG126712	AB847658	AB847790
*D. laevifolium*	*Oxyglossum*	TBG142428	AB593593	AB847791
*D. lamyaiae*	*Dendrobium*	TBG116046	AB593595	AB847792
*D. lancifolium*	*Calcarifera*	TBG142427	AB847659	AB847793
*D. laterale*	*Stachyobium*	TNS8501719	AB847660	AB847794
*D. lawesii*	*Calyptrochilus*	TBG140275	AB593596	AB847795
*D. leonis*	*Aporum*	84206	AB593597	AB847796
*D. leptocladum*	*Dendrobium*	TNS8502939	AB593598	AB847797
*D. linawianum*	*Dendrobium*	TBG142429	AB593599	AB847798
*D. lindleyi*	*Densiflora*	TBG115834	AB593600	AB847799
*D. linguella*	*Breviflores*	TBG115831	AB593601	AB847800
*D. lituiflorum*	*Dendrobium*	TBG128908	AB593602	AB847801
*D. lobbii*	*Conostalix*	NSW426014	AB593603	AB847802
*D. loddigesii*	*Dendrobium*	74531	AB593604	AB847803
*D. lohohense*	*Holochrysa*	TBG132863	AB593605	AB847804
*D. longicornu*	*Formosae*	TBG122802	AB847661	AB847805
*D. lowii*	*Formosae*	TBG134848	AB593606	AB847806
*D. luteolum*	*Dendrobium*	TBG122798	AB593607	AB847807
*D. maccarthiae*	*Dendrobium*	TBG128803	AB593608	AB847808
*D. macfarlanei*	*Crumenata*	TBG122496	AB847662	AB847809
*D. macrostachyum*	*Dendrobium*	TBG123340	AB847663	AB847810
*D. masarangense* subsp. *theionanthum*	*Oxyglossum*	Tajima 27	AB593609	AB847811
*D. melanostictum*	*Amblyanthus*	TBG123873	AB593610	AB847812
*D. melinanthum*	*Calyptrochilus*	TBG159436	AB593611	AB847813
*D. microglaphys*	*Amblyanthus*	TBG135481	AB593612	AB847814
*D. mohlianum*	*Calyptrochilus*	TBG124783	AB593613	AB847815
*D. moniliforme*	*Dendrobium*	TBG115845	AB593614	AB847816
*D. morrisonii*	*Pedilonum*	Yukawa 97-2045	AB847664	AB847817
*D. moschatum*	*Holochrysa*	56113	AB593616	AB847818
*D. mutabile*	*Calcarifera*	TBG159437	AB593617	AB847819
*D. nemorale*	*Distichophyllae*	TBG159440	AB593618	AB847820
*D. nobile*	*Dendrobium*	TBG128809	AB593619	AB847821
*D. ochreatum*	*Dendrobium*	TBG128865	AB593621	AB847822
*D. okinawense*	*Dendrobium*	TBG115938	AB593622	AB847823
*D. oligophyllum*	*Distichophyllae*	TBG128728	AB847665	AB847824
*D. pachyglossum*	*Conostalix*	TBG124380	AB593623	AB847825
*D. pachyphyllum*	*Bolbodium*	TBG126623	AB593624	AB847826
*D. palpebrae_*1	*Densiflora*	TBG159441	AB593625	AB847827
*D. palpebrae_*2	*Densiflora*	TBG159442	AB593626	AB847828
*D. palpebrae_*3	*Densiflora*	TBG118313	AB593627	AB847829
*D. panduriferum*	*Calcarifera*	TBG144531	AB847666	AB847830
*D. papilio*	*Dolichocentrum*	TBG120493	AB847667	AB847831
*D. parciflorum*	*Aporum*	TBG118261	AB593628	AB847832
*D. parcum*	*Herbaceae*	TBG129884	AB593629	AB847833
*D. parishii*	*Dendrobium*	TBG159443	AB593630	AB847834
*D. parthenium*	*Formosae*	TBG136015	AB847668	AB847835
*D. patentilobum*	*Aporum*	TBG137060	AB847669	AB847836
*D. pendulum*	*Dendrobium*	TBG128867	AB593633	AB847837
*D. philippinense*	*Crumenata*	TBG142250	AB593634	AB847838
*D. platygastrium*	*Platycaulon*	TBG142432	AB593635	AB847839
*D. polyanthum*	*Dendrobium*	TBG159417	AB593636	AB847840
*D. porphyrochilum*	*Stachyobium*	TBG141468	AB593637	AB847841
*D. praecinctum*	*Stuposa*	TBG116030	AB593638	AB847842
*D. prasinum*	*Oxyglossum*	TBG119108	AB593639	AB847843
*D. prianganense*	*Calcarifera*	TBG134835	AB593640	AB847844
*D. primulinum*	*Dendrobium*	TBG159445	AB593641	AB847845
*D. profusum*	*Calcarifera*	TBG133266	AB593642	AB847846
*D. pulchellum*	*Holochrysa*	TBG118088	AB593643	AB847847
*D. rarum*	*Pedilonum*	TBG124851	AB593644	AB847848
*D. regium*	*Dendrobium*	TNS8501314	AB593645	AB847849
*D. rhombeum_*1	*Dendrobium*	TBG135976	AB593646	AB847850
*D. rhombeum_*2	*Dendrobium*	TBG142436	AB593647	AB847851
*D. roseiodorum*	*Formosae*	TBG118270	AB593648	AB847852
*D. roseipes*	*Pedilonum*	TBG142437	AB593649	AB847853
*D. rosellum*	*Aporum*	TBG133320	AB593650	AB847854
*D. ruckeri*	*Dendrobium*	TBG159446	AB593651	AB847855
*D. sanderae*	*Formosae*	TBG120503	AB593654	AB847856
*D. sanguinolentum*	*Calcarifera*	TBG159447	AB593655	AB847857
*D. scabrilingue*	*Formosae*	TBG119087	AB593656	AB847858
*D. schuetzei*	*Formosae*	TBG119088	AB593658	AB847859
*D. scoriarum*	*Dendrobium*	YukawaDNA0381	AB593659	AB847860
*D. secundum*	*Pedilonum*	TBG100260	AB593660	AB847862
*D. senile*	*Dendrobium*	TBG119090	AB593661	AB847863
*D. signatum*	*Dendrobium*	TBG133598	AB593662	AB847864
*D. singkawangense*	*Formosae*	TBG141081	AB593663	AB847865
*D. smillieae*	*Pedilonum*	TBG1402699	AB593664	AB847866
*D.* sp.	*Stachyobium*	YukawaDNA0749	AB847670	AB847861
*D. spatella*	*Aporum*	TBG84203	AB847671	AB847867
*D. speckmaieri*	*Platycaulon*	TBG136027	AB593665	AB847868
*D. spectatissimum*	*Formosae*	TBG133877	AB593666	AB847869
*D. stockeri*	*Amblyanthus*	TBG130082	AB593667	AB847870
*D. stuposum*	*Stuposa*	TBG134843	AB593668	AB847871
*D. sulcatum*	*Densiflora*	TBG159451	AB593670	AB847872
*D. sutepense*	*Formosae*	TNS8503543	AB593671	AB847873
*D. suzukii*	*Formosae*	TNS9518319	AB593672	AB847874
*D. tetrachromum*	*Dendrobium*	TBG137068	AB847672	AB847875
*D. thyrsiflorum*	*Densiflora*	TBG159453	AB593674	AB847876
*D. tobaense*	*Formosae*	TBG126681	AB593677	AB847877
*D. tortile*	*Dendrobium*	TBG119099	AB593678	AB847878
*D. trankimianum*	*Formosae*	TBG127512	AB847673	AB847879
*D. transparens*	*Dendrobium*	TBG126731	AB593679	AB847880
*D. trantuanii*	*Breviflores*	TBG157296	AB847674	AB847881
*D. trichostomum*	*Calyptrochilus*	TBG142265	AB593680	AB847882
*D. trigonopus*	*Formosae*	TBG119100	AB847675	AB847883
*D. unicum*	*Dendrobium*	TBG119101	AB593682	AB847884
*D. uniflorum*	*Distichophyllae*	TBG120499	AB593683	AB847885
*D. venustum*	*Stachyobium*	TBG129885	AB847676	AB847886
*D. victoriae-reginae*	*Calcarifera*	TBG141156	AB593684	AB847887
*D. violaceum*	*Oxyglossum*	TBG100203	AB593685	AB847888
*D. wardianum*	*Dendrobium*	TBG126641	AB593686	AB847889
*D. ypsilon*	*Platycaulon*	TBG118514	AB593688	AB847890
