# Supplementary material for: Molecular phylogenetics and character evolution of morphologically diverse groups, Dendrobium section Dendrobium and allies
Source: AoB Plants. 2014 Aug 7;6:plu045. doi: 10.1093/aobpla/plu045 (PMC4172198; doi:10.1093/aobpla/plu045)
Supplement: Additional Information [file supp_6_plu045_index.html]

Molecular phylogenetics and character evolution of morphologically diverse groups, Dendrobium section Dendrobium and allies — Additional Information 

# Molecular phylogenetics and character evolution of morphologically diverse groups, *Dendrobium* section *Dendrobium* and allies

## Additional Information

Additional Information

**Files in this Data Supplement:**

- Additional Information - Pdf file
