# Supplementary material for: Molecular phylogenetics and character evolution of morphologically diverse groups, Dendrobium section Dendrobium and allies
Source: AoB Plants. 2014 Aug 7;6:plu045. doi: 10.1093/aobpla/plu045 (PMC4172198; doi:10.1093/aobpla/plu045)
Supplement: Additional Information [file supp_plu045_plu045supp.pdf]

SUPPLEMENTARY DATA

FIG. S1-1. Consensus phylogram obtained from 100754 bayesian trees with higher posterior probability from ITS sequence

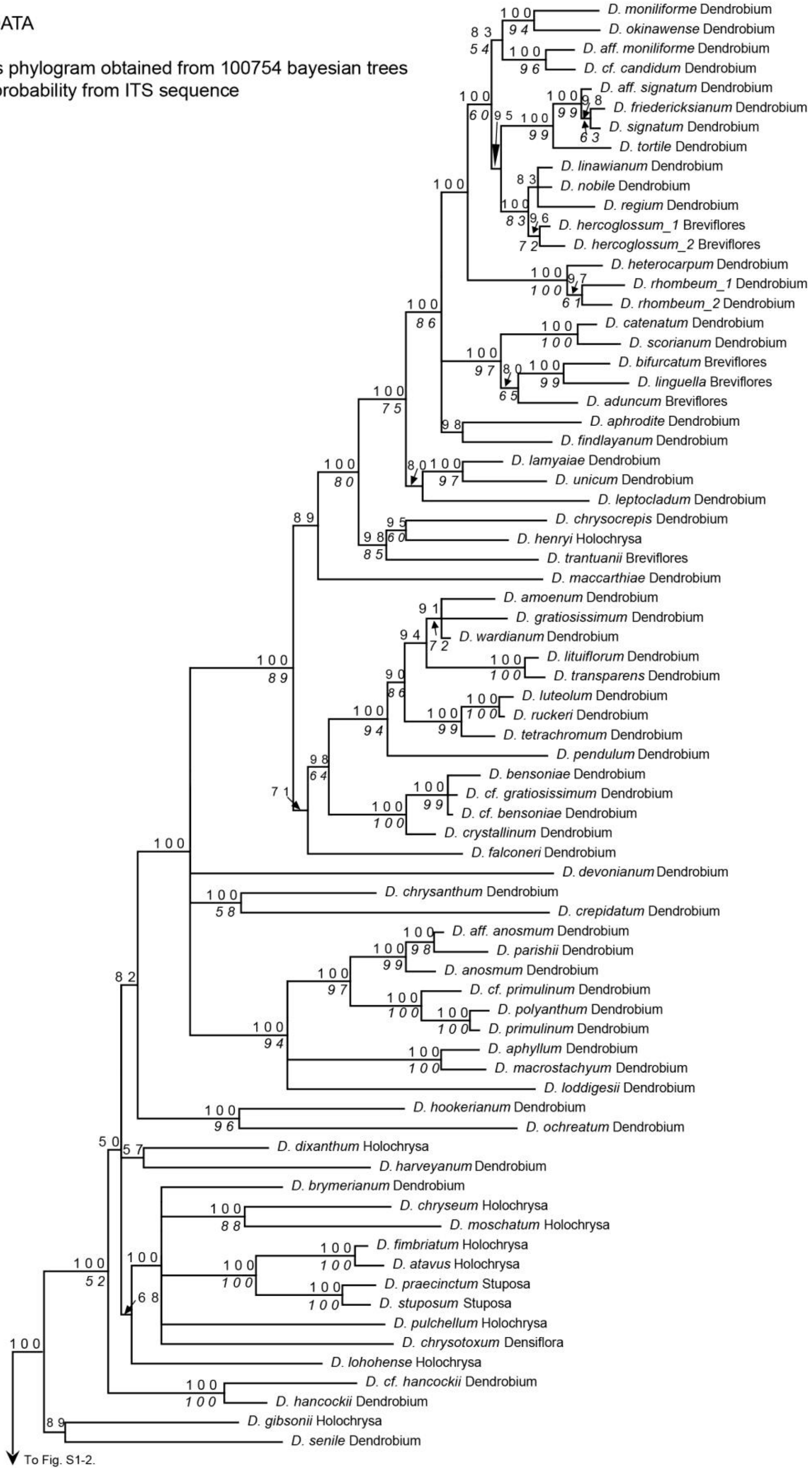

FIG. S1-2.

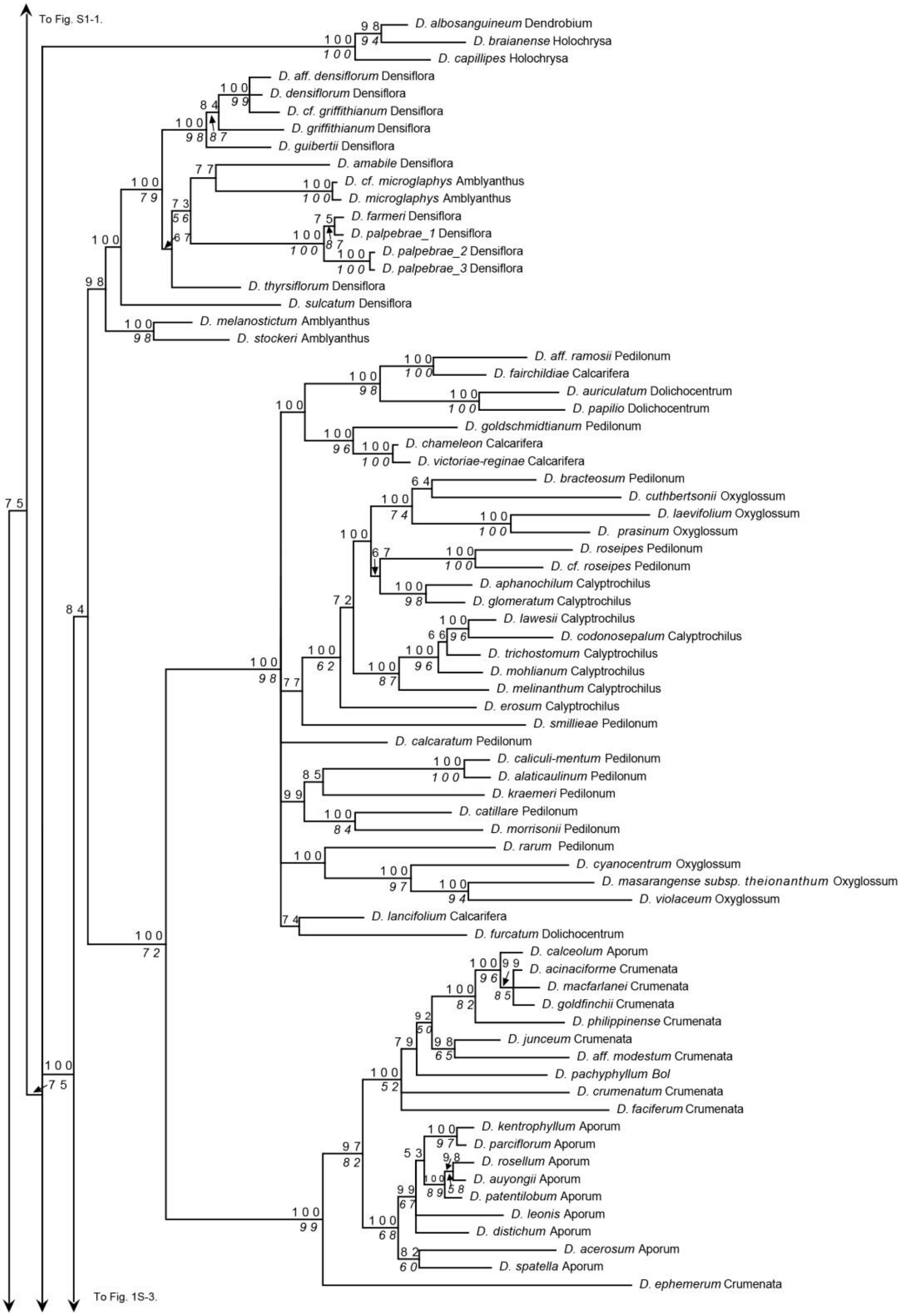
To Fig. S1-1.
D. albosanguineum Dendrobium
D. braianense Holochrysa
D. capillipes Holochrysa
D. aff. densiflorum Densiflora
D. densiflorum Densiflora
D. cf. griffithianum Densiflora
D. griffithianum Densiflora
D. guibertii Densiflora
D. amabile Densiflora
D. cf. microglaphys Amblyanthus
D. microglaphys Amblyanthus
D. farmeri Densiflora
D. palpebrae_1 Densiflora
D. palpebrae_2 Densiflora
D. palpebrae_3 Densiflora
D. thyrsiflorum Densiflora
D. sulcatum Densiflora
D. melanostictum Amblyanthus
D. stockeri Amblyanthus
D. aff. ramosii Pedilonum
D. fairchildiae Calcarifera
D. auriculatum Dolichocentrum
D. papilio Dolichocentrum
D. goldschmidtianum Pedilonum
D. chameleon Calcarifera
D. victoriae-reginae Calcarifera
D. bracteosum Pedilonum
D. cuthbertsonii Oxyglossum
D. laevifolium Oxyglossum
D. prasinum Oxyglossum
D. roseipes Pedilonum
D. cf. roseipes Pedilonum
D. aphanochilum Calyptrochilus
D. glomeratum Calyptrochilus
D. lawesii Calyptrochilus
D. codonosepalum Calyptrochilus
D. trichostomum Calyptrochilus
D. mohlianum Calyptrochilus
D. melinanthum Calyptrochilus
D. erosum Calyptrochilus
D. smillieae Pedilonum
D. calcaratum Pedilonum
D. caliculi-mentum Pedilonum
D. alaticaulinum Pedilonum
D. kraemeri Pedilonum
D. catillare Pedilonum
D. morrisonii Pedilonum
D. rarum Pedilonum
D. cyanocentrum Oxyglossum
D. masarangense subsp. theionanthum Oxyglossum
D. violaceum Oxyglossum
D. lancifolium Calcarifera
D. furcatum Dolichocentrum
D. calceolum Aporum
D. acinaciforme Crumenata
D. macfarlanei Crumenata
D. goldfinchii Crumenata
D. philippinense Crumenata
D. junceum Crumenata
D. aff. modestum Crumenata
D. pachyphyllum Bol
D. crumenatum Crumenata
D. faciferum Crumenata
D. kentrophyllum Aporum
D. parciflorum Aporum
D. rosellum Aporum
D. auyongii Aporum
D. patentilobum Aporum
D. leonis Aporum
D. distichum Aporum
D. acerosum Aporum
D. spatella Aporum
D. ephemerum Crumenata
To Fig. 1S-3.

FIG. S1-3.

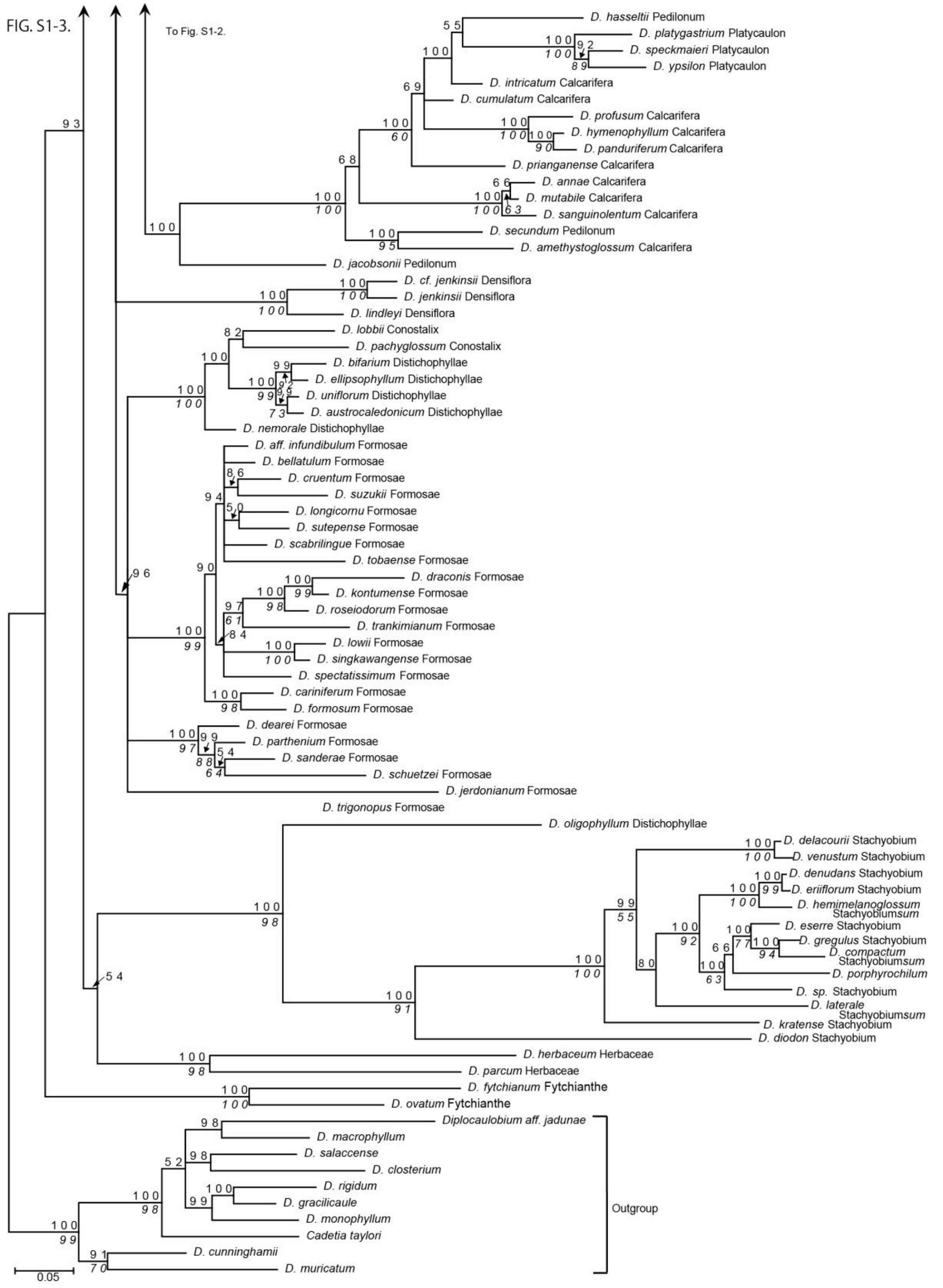
To Fig. S1-2.
D. hasseltii Pedilonum
D. platygastrium Platycaulon
D. speckmaieri Platycaulon
D. ypsilon Platycaulon
D. intricatum Calcarifera
D. cumulatum Calcarifera
D. profusum Calcarifera
D. hymenophyllum Calcarifera
D. panduriferum Calcarifera
D. prianganense Calcarifera
D. annae Calcarifera
D. mutabile Calcarifera
D. sanguinolentum Calcarifera
D. secundum Pedilonum
D. amethystoglossum Calcarifera
D. jacobsonii Pedilonum
D. cf. jenkinsii Densiflora
D. jenkinsii Densiflora
D. lindleyi Densiflora
D. lobbii Conostalix
D. pachyglossum Conostalix
D. bifarium Distichophyllae
D. ellipsophyllum Distichophyllae
D. uniflorum Distichophyllae
D. austrocaledonicum Distichophyllae
D. nemorale Distichophyllae
D. aff. infundibulum Formosae
D. bellatulum Formosae
D. cruentum Formosae
D. suzukii Formosae
D. longicornu Formosae
D. sutepense Formosae
D. scabrilingue Formosae
D. tobaense Formosae
D. draconis Formosae
D. kontumense Formosae
D. roseiodorum Formosae
D. trankimianum Formosae
D. lowii Formosae
D. singkawangense Formosae
D. spectatissimum Formosae
D. cariniferum Formosae
D. formosum Formosae
D. dearei Formosae
D. parthenium Formosae
D. sanderae Formosae
D. schuetzei Formosae
D. jerdonianum Formosae
D. trigonopus Formosae
D. oligophyllum Distichophyllae
D. delacourii Stachyobium
D. venustum Stachyobium
D. denudans Stachyobium
D. eriiflorum Stachyobium
D. hemimelanoglossum Stachyobiumsum
D. eserre Stachyobium
D. gregulus Stachyobium
D. compactum Stachyobiumsum
D. porphyrochilum
D. sp. Stachyobium
D. laterale Stachyobiumsum
D. kratense Stachyobium
D. diodon Stachyobium
D. herbaceum Herbaceae
D. parcum Herbaceae
D. fytchianum Fytchianthe
D. ovatum Fytchianthe
Diplocaulobium aff. jadunae
D. macrophyllum
D. salaccense
D. closterium
D. rigidum
D. gracilicaule
D. monophyllum
Cadetia taylori
D. cunninghamii
D. muricatum
Outgroup
0.05

SUPPLEMENTARY DATA

FIG. S2-1. Consensus phylogram obtained from 119935 bayesian trees with higher posterior probability from *matK* sequence

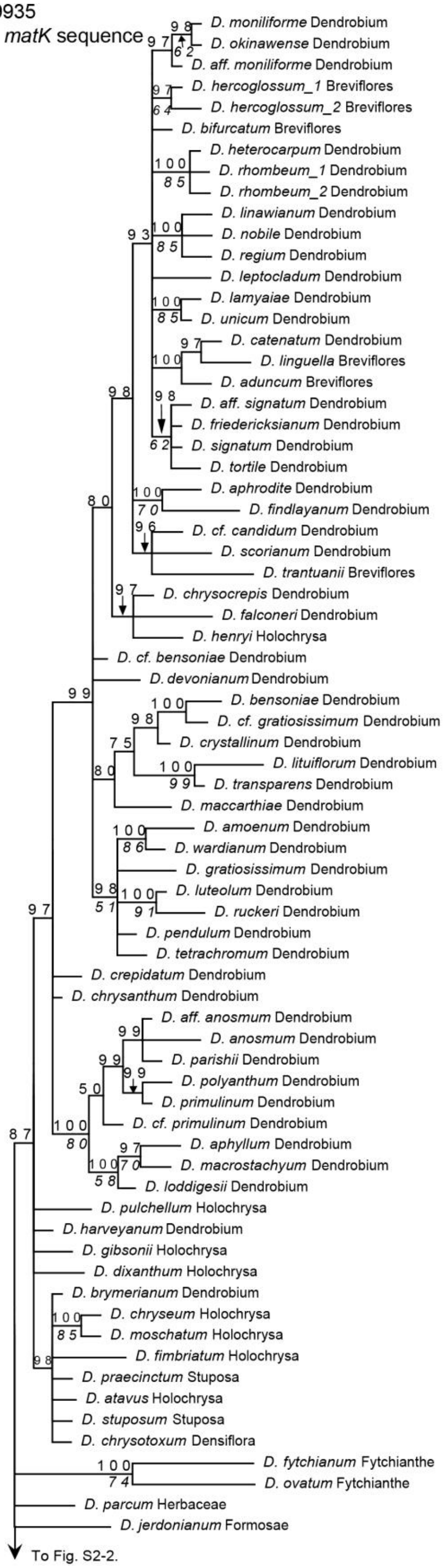

FIG. S2-2.

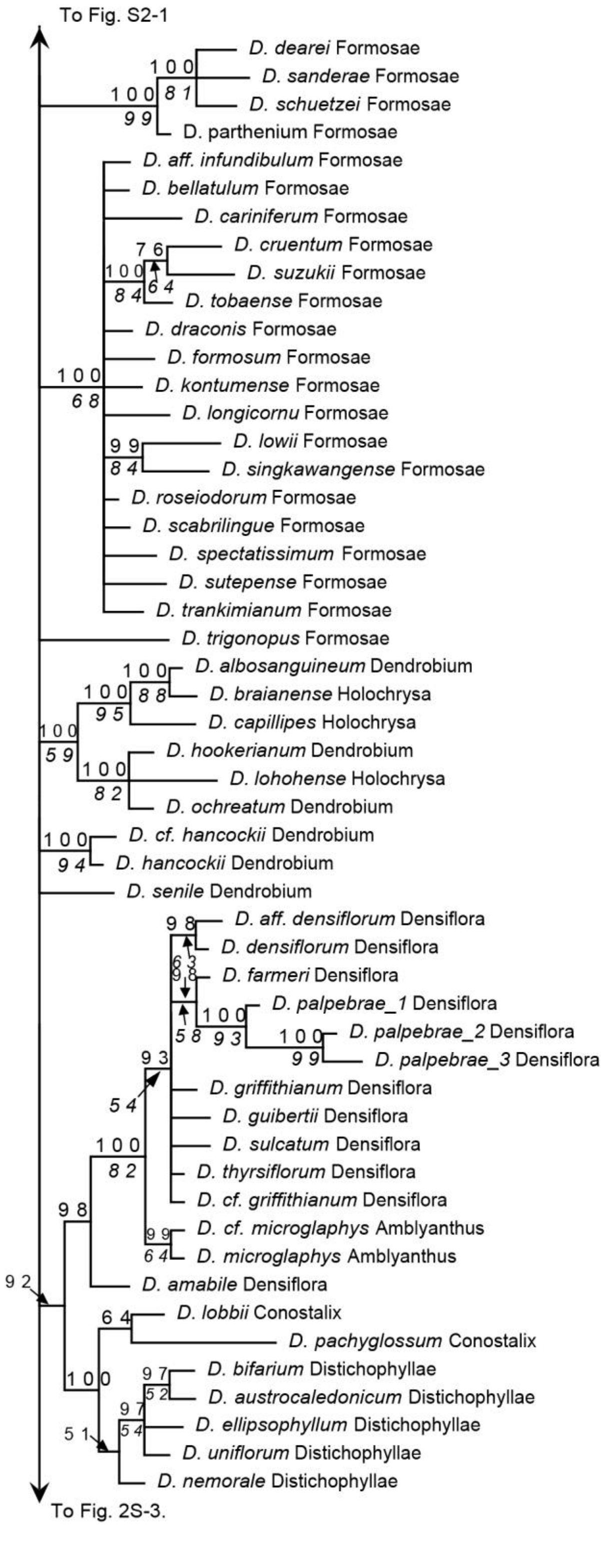
To Fig. S2-1
D. dearei Formosae
D. sanderae Formosae
D. schuetzei Formosae
D. parthenium Formosae
D. aff. infundibulum Formosae
D. bellatulum Formosae
D. cariniferum Formosae
D. cruentum Formosae
D. suzukii Formosae
D. tobaense Formosae
D. draconis Formosae
D. formosum Formosae
D. kontumense Formosae
D. longicornu Formosae
D. lowii Formosae
D. singkawangense Formosae
D. roseiodorum Formosae
D. scabrilingue Formosae
D. spectatissimum Formosae
D. sutepense Formosae
D. trankimianum Formosae
D. trigonopus Formosae
D. albosanguineum Dendrobium
D. braianense Holochrysa
D. capillipes Holochrysa
D. hookerianum Dendrobium
D. lohohense Holochrysa
D. ochreatum Dendrobium
D. cf. hancockii Dendrobium
D. hancockii Dendrobium
D. senile Dendrobium
D. aff. densiflorum Densiflora
D. densiflorum Densiflora
D. farmeri Densiflora
D. palpebrae_1 Densiflora
D. palpebrae_2 Densiflora
D. palpebrae_3 Densiflora
D. griffithianum Densiflora
D. guibertii Densiflora
D. sulcatum Densiflora
D. thyrsiflorum Densiflora
D. cf. griffithianum Densiflora
D. cf. microglaphys Amblyanthus
D. microglaphys Amblyanthus
D. amabile Densiflora
D. lobbii Conostalix
D. pachyglossum Conostalix
D. bifarium Distichophyllae
D. austrocaledonicum Distichophyllae
D. ellipsophyllum Distichophyllae
D. uniflorum Distichophyllae
D. nemorale Distichophyllae
To Fig. 2S-3.

FIG. S2-3.

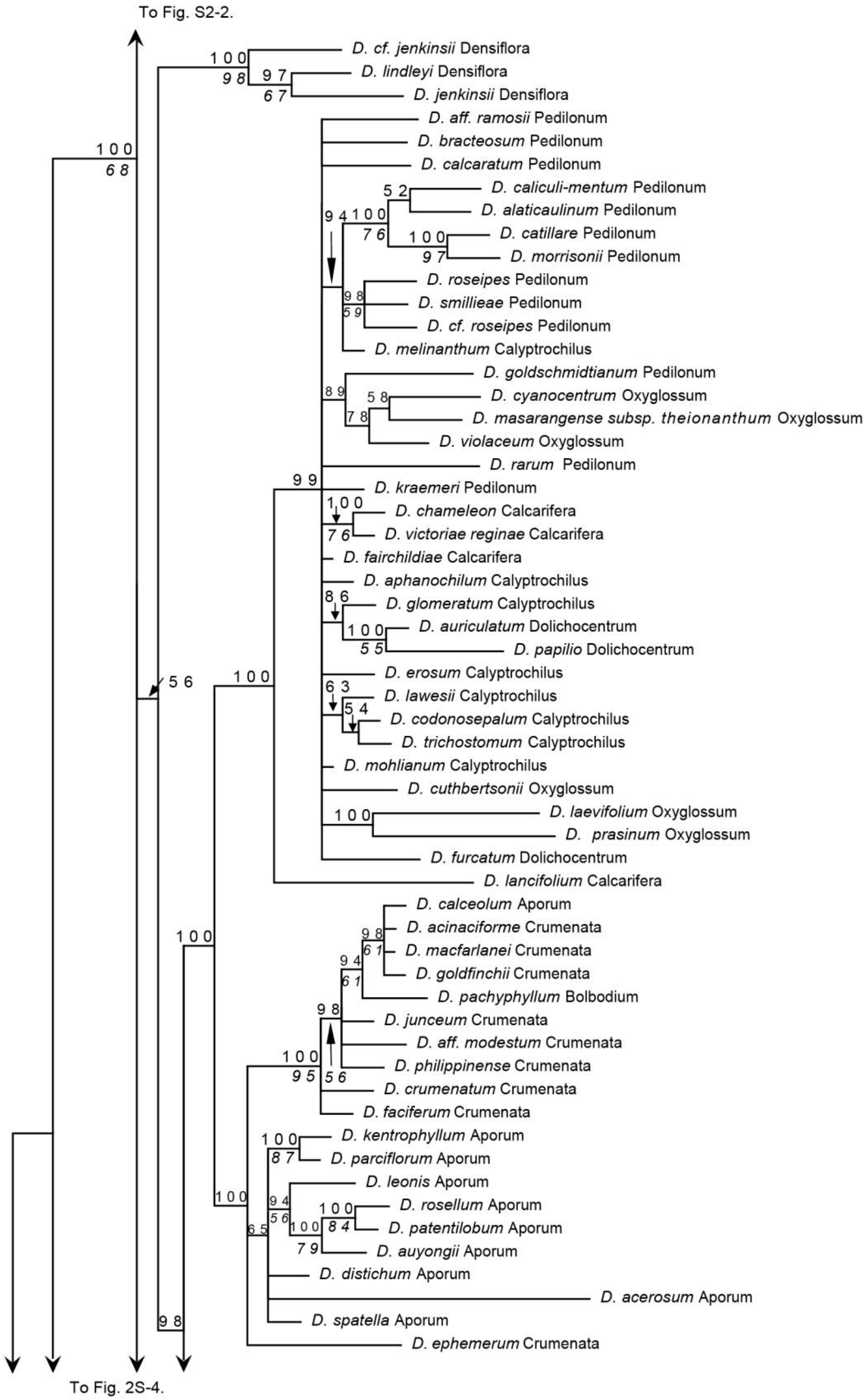
To Fig. S2-2.
D. cf. jenkinsii Densiflora
D. lindleyi Densiflora
D. jenkinsii Densiflora
D. aff. ramosii Pedilonum
D. bracteosum Pedilonum
D. calcaratum Pedilonum
D. caliculi-mentum Pedilonum
D. alaticaulinum Pedilonum
D. catillare Pedilonum
D. morrisonii Pedilonum
D. roseipes Pedilonum
D. smillieae Pedilonum
D. cf. roseipes Pedilonum
D. melinanthum Calyptrochilus
D. goldschmidtianum Pedilonum
D. cyanocentrum Oxyglossum
D. masarangense subsp. theionanthum Oxyglossum
D. violaceum Oxyglossum
D. rarum Pedilonum
D. kraemeri Pedilonum
D. chameleon Calcarifera
D. victoriae reginae Calcarifera
D. fairchildiae Calcarifera
D. aphanochilum Calyptrochilus
D. glomeratum Calyptrochilus
D. auriculatum Dolichocentrum
D. papilio Dolichocentrum
D. erosum Calyptrochilus
D. lawesii Calyptrochilus
D. codonosepalum Calyptrochilus
D. trichostomum Calyptrochilus
D. mohlianum Calyptrochilus
D. cuthbertsonii Oxyglossum
D. laevifolium Oxyglossum
D. prasinum Oxyglossum
D. furcatum Dolichocentrum
D. lancifolium Calcarifera
D. calceolum Aporum
D. acinaciforme Crumenata
D. macfarlanei Crumenata
D. goldfinchii Crumenata
D. pachyphyllum Bolbodium
D. junceum Crumenata
D. aff. modestum Crumenata
D. philippinense Crumenata
D. crumenatum Crumenata
D. faciferum Crumenata
D. kentrophyllum Aporum
D. parciflorum Aporum
D. leonis Aporum
D. rosellum Aporum
D. patentilobum Aporum
D. auyongii Aporum
D. distichum Aporum
D. acerosum Aporum
D. spatella Aporum
D. ephemerum Crumenata
To Fig. 2S-4.

FIG. S2-4.

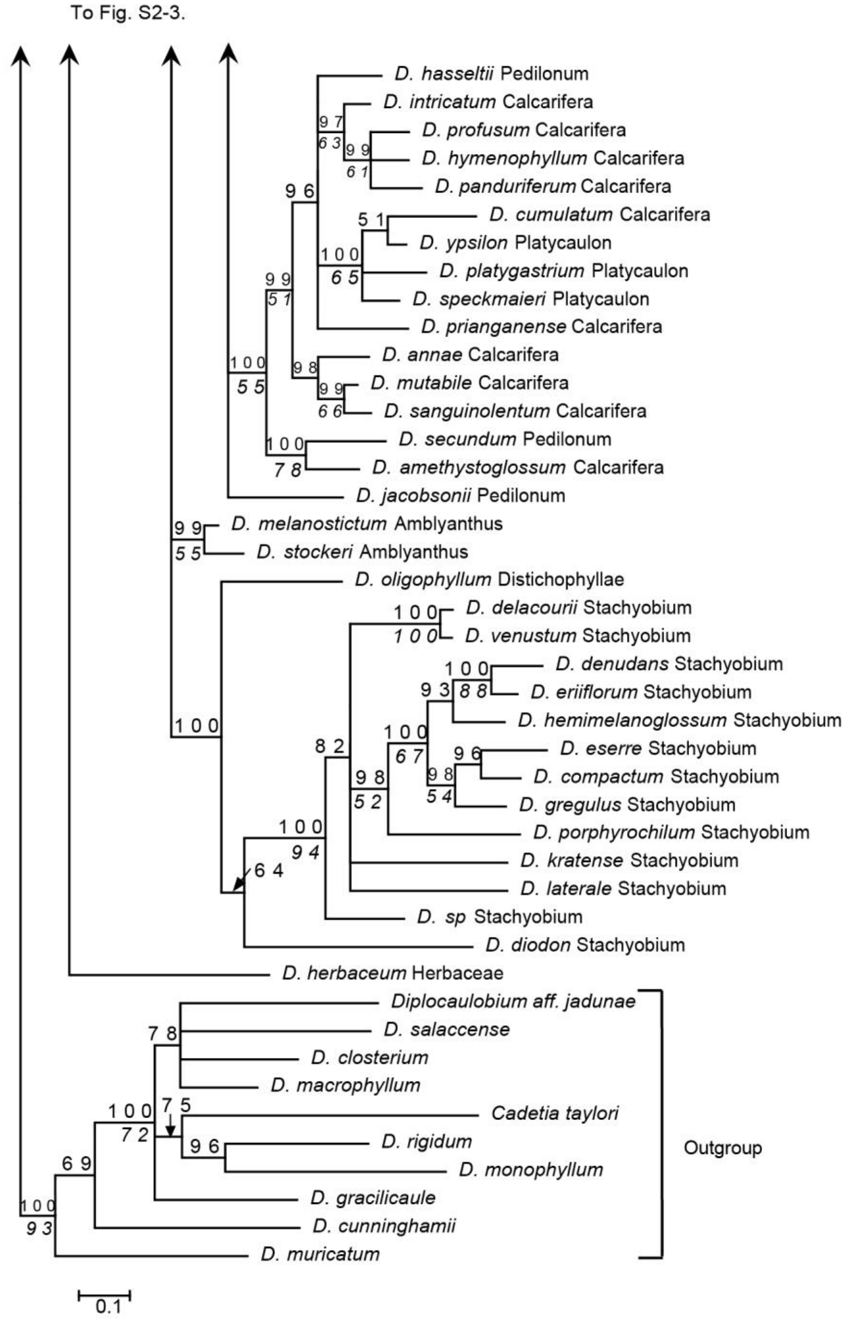
To Fig. S2-3.
D. hasseltii Pedilonum
D. intricatum Calcarifera
D. profusum Calcarifera
D. hymenophyllum Calcarifera
D. panduriferum Calcarifera
D. cumulatum Calcarifera
D. ypsilon Platycaulon
D. platygastrium Platycaulon
D. speckmaieri Platycaulon
D. prianganense Calcarifera
D. annae Calcarifera
D. mutabile Calcarifera
D. sanguinolentum Calcarifera
D. secundum Pedilonum
D. amethystoglossum Calcarifera
D. jacobsonii Pedilonum
D. melanostictum Amblyanthus
D. stockeri Amblyanthus
D. oligophyllum Distichophyllae
D. delacourii Stachyobium
D. venustum Stachyobium
D. denudans Stachyobium
D. eriiflorum Stachyobium
D. hemimelanoglossum Stachyobium
D. eserre Stachyobium
D. compactum Stachyobium
D. gregulus Stachyobium
D. porphyrochilum Stachyobium
D. kratense Stachyobium
D. laterale Stachyobium
D. sp Stachyobium
D. diodon Stachyobium
D. herbaceum Herbaceae
Diplocaulobium aff. jadunae
D. salaccense
D. closterium
D. macrophyllum
Cadetia taylori
D. rigidum
D. monophyllum
D. gracilicaule
D. cunninghamii
D. muricatum
Outgroup
0.1
